# Association between child maltreatment and depressive symptoms in emerging adulthood: The mediating and moderating roles of DNA methylation

**DOI:** 10.1371/journal.pone.0280203

**Published:** 2023-01-12

**Authors:** Maude Comtois-Cabana, Emily Barr, Nadine Provençal, Isabelle Ouellet-Morin

**Affiliations:** 1 Department of Psychology, University of Montreal, Montreal, Quebec, Canada; 2 Faculty of Health Sciences, Simon Fraser University, Burnaby, British Columbia, Canada; 3 BC Children’s Hospital Research Institute, Vancouver, British Columbia, Canada; 4 School of Criminology, University of Montreal, Montreal, Quebec, Canada; 5 Research Center of the Montreal Mental Health University Institute, Montreal, Quebec, Canada; University of Pavia: Universita degli Studi di Pavia, ITALY

## Abstract

Prospective studies suggest that child maltreatment substantially increases the risk for depression in adulthood. However, the mechanisms underlying this association require further elucidation. In recent years, DNA methylation has emerged as a potential mechanism by which maltreatment experiences (a) could partly explain the emergence or aggravation of depressive symptoms (i.e., mediation) and/or (b) could increase (or decrease) the risk for depressive symptoms (i.e., moderation). The present study tested whether the methylation levels of nine candidate genes mediated and/or moderated the association between maltreatment experiences in childhood and depressive symptoms in emerging adulthood. The sample comprised 156 men aged between 18 and 35 years. Maltreatment experiences and depressive symptoms were assessed retrospectively using self-reported questionnaires. Methylation levels of nine candidate genes (*COMT*, *FKBP5*, *IL6*, *IL10*, *MAOA*, *NR3C1*, *OXTR*, *SLC6A3* and *SLC6A4*), previously reported to be sensitive to early-life stress, were quantified from saliva samples. Maltreatment experiences in childhood were significantly associated with depressive symptoms in emerging adulthood. Both maltreatment experiences and depressive symptoms were associated with the methylation levels of two genomic sites, which cumulatively, but not individually, explained 16% of the association between maltreatment experiences in childhood and depressive symptoms in emerging adulthood. Moreover, maltreatment experiences in childhood interacted with the methylation levels of fourteen genomic sites, which cumulatively, but not individually, modulated the level of depressive symptoms in young male adults who were maltreated as children. However, none of these effects survived multiple testing correction. These findings bring attention to the cumulative effects of DNA methylation measured in several candidate genes on the risk of reporting depressive symptoms following maltreatment experiences in childhood. Nonetheless, future studies need to clarify the robustness of these putative cumulative effects in larger samples and longitudinal cohorts.

## Introduction

Prospective studies suggest that child maltreatment substantially increases the risk for depression in adulthood [1]. Moreover, the global prevalence of self-reported maltreatment experiences are 12.7% for sexual abuse, 22.6% for physical abuse, 36.3% for emotional abuse, 16.3% for physical neglect, and 18.4% for emotional neglect [2]. Therefore, it is imperative to better understand how child maltreatment is associated with higher levels of depressive symptoms (i.e., mediating factors), as well as in which contexts or for whom these symptoms are more likely to arise following these experiences (i.e., moderating factors). In recent years, epigenetic marks have emerged as potential mediating and moderating factors of the association between early-life stress and psychopathology [3].

The most commonly studied epigenetic mark is DNA methylation (DNAm), which refers to the addition of a methyl group (-CH_3_) onto a cytosine base followed by a guanine base (i.e., CpG site) [4]. This epigenetic mark prevents gene transcription in two ways: (a) methylated CpG sites impede the binding of transcriptional activators and (b) methylated CpG sites promote the binding of transcriptional repressors [5]. In general, methylated CpG sites that are located within the promoter region of a gene are associated with decreased expression of that gene [6]. Notably, these marks can be maintained through cell division, which may induce persistent changes in gene activity and, thus, may lead to stable alterations in biological processes. In addition to being responsive to the genome [7], DNAm profiles are also responsive to environmental exposures, both physical and social [8].

A compelling body of theory and research suggests that exposure to stressful experiences, such as child maltreatment, may become ‘biologically embedded’. Biological embedding is the concept that the exposure to stressful experiences during sensitive periods of development engenders lasting biological changes, altering the development and functioning of stress-related neurobiological systems, and therefore contributing to the emergence of physical and mental health problems later in life [9]. This concept is supported, for example, by studies reporting atypical cortisol responses to stress in adults maltreated as children [10, 11]. Despite increasing knowledge of the neurobiological consequences of early-life stress, the molecular mechanisms underlying the biological embedding of these experiences requires further elucidation. In recent years, DNAm has emerged as a promising mechanism driving biological embedding [12].

Although epigenome-wide association studies (i.e., EWAS) are now preferred, many studies investigating the associations between maltreatment experiences, DNAm levels and depressive symptoms employed a candidate gene approach, whereby genes are pre-selected on the basis of prior knowledge regarding their neurobiological functions in terms of sensitivity to stress and/or the pathogenesis of depression, such as genes involved in neuroendocrine (*FKBP5*, *NR3C1*, *OXTR*), neurotransmitters (*COMT*, *MAOA*, *SLC6A3*, *SLC6A4*) and inflammatory pathways (*IL6*, *IL10*). Cecil et al. [13] and Parade et al. [14] reported that although maltreatment experiences were associated with DNAm levels, the results were often mixed between studies targeting a same gene. To date, the most solid evidence emerges for the *NR3C1* gene, which encodes glucocorticoid receptors, with most studies reporting higher DNAm levels in participants with a history of child maltreatment [13–15]. The robustness of this association is noteworthy; as noted across species (e.g., [16]), developmental stages (childhood [e.g., [17]], adolescence [e.g., [18]], adulthood [e.g., [19]]), samples (clinical [e.g., [20]], community [e.g., [21]]), tissues (blood [e.g., [22]], brain [e.g., [23, 24]], saliva [e.g., [25]]), maltreatment measures (self-report [e.g., [26]], official records [e.g., [27]]), designs (retrospective [e.g., [28]], prospective [e.g., [29]] and approaches (EWAS [e.g., [30]]). However, this has not been the case for other candidate genes involved in the neuroendocrine response to stress. Studies on *FKBP5*, which encodes proteins that regulate the sensitivity of glucocorticoid receptors to cortisol, reported either negative (e.g., [31]) or no association (e.g., [32]) between child maltreatment and DNAm levels, while studies on *OXTR*, which encodes oxytocin receptors, reported no overall association (e.g., [33]). The maltreatment literature also focused on genes involved in the serotoninergic and dopaminergic pathways, such as *SLC6A4*, which encodes serotonin transporters responsible for serotonin reuptake, and *MAOA*, which encodes enzymes involved in the degradation of dopamine and serotonin. Studies on *SLC6A4* reported positive (e.g., [34]) or no associations (e.g., [35]) between maltreatment experiences and DNAm levels, while studies on *MAOA* reported positive associations (e.g., [36]) or no associations (e.g., [37]). That is, important methodological constraints currently limit the conclusions that can be drawn from existing DNAm studies in the context of maltreatment, such as the lack of control for well-known DNAm confounders and multiple testing [13, 14]. Cecil et al. [13] also suggested to use continuous assessments of child maltreatment to capture a wide range of exposures and to increase statistical power, as well as to measure all types of maltreatment to account for their potential cumulative effects.

Regarding the depression literature, Li et al. [38] reported in their recent systematic review that although depressive symptoms were associated with DNAm levels, the strength and/or direction of this association varied dramatically across studies. To date, however, and despite considerable differences in study designs (e.g., sample size, sample characteristics, biological samples), laboratory techniques (e.g., DNA extraction methods/kits, DNA methylation methods/kits) and statistical analyses (e.g., parametric models, non-parametric models), the most solid evidence points to the *SLC6A4* gene, for which most studies reported higher DNAm levels in depressed participants compared to their healthy counterparts [38]. However, conflicting results (i.e., both higher and lower DNAm levels or non-significant differences) were reported in the few studies that focused on the *FKBP5*, *MAOA*, *NR3C1* and *OXTR* genes [38]. For example, in adult samples, Melas et al. [39] detected higher *NR3C1* DNAm levels in saliva samples, while Na et al. [40] detected lower *NR3C1* DNAm levels in blood samples levels, whereas Alt et al. [41] reported no differences in brain samples. Although most studies focused on case-control differences, depression is a continuous measure that captures both clinical and sub-clinical depressive symptoms, for which associated DNAm signatures may signal a risk for or a consequence of a depressive symptomatology [42].

Altogether, existing studies seem to support the hypothesis that DNAm may partly explain the association between maltreatment experiences in childhood and depressive symptoms in adulthood. However, to this day, very few studies have formally tested the presumed mediating role of DNAm, which requires going beyond the investigation of the bivariate associations between maltreatment experiences, methylation levels, and depressive symptoms, and to directly estimate the significance of the indirect effect of DNAm linking maltreatment experiences to depressive symptoms. For instance, Bustamante et al. [32] found that *FKBP5* methylation levels did not explain the association between child maltreatment and the severity of depressive symptoms among adults. Similarly, Smearman et al. [43] found that *OXTR* methylation levels did not mediate the association between child maltreatment and depression and anxiety symptoms in adulthood. In contrast, Checknita et al. [36] found that *MAOA* methylation levels mediated the association between sexual abuse and depression. Interestingly, Peng et al. [37] found that the methylation levels of two CpG sites in *BDNF* and *NR3C1* explained about 20% of the association between traumatic experiences in childhood and depressive symptoms in adulthood. In sum, although theoretical frameworks suggest that DNAm could partially explain higher risk of depressive symptoms following maltreatment experiences, empirical evidence is still limited, and so far, rather inconsistent.

DNAm has also been hypothesized to modulate the influence that social environments may exert on the development of mental health problems [44]. This hypothesis is consistent with the idea that the extent to which child maltreatment is associated with depressive symptoms may differ across individuals and contexts, which is shared by several stress models, such as the diathesis-stress [45] and the differential susceptibility [46] models. However, to this day, very few studies have formally tested the presumed moderating role of DNAm. For instance, Radtke et al. [28] found that the methylation levels of nine CpG sites in *NR3C1* moderated the association between maltreatment experiences in childhood and psychopathological symptoms in adulthood, but the direction of the interaction is not specified. Similarly, Smearman et al. [43] found that the methylation levels of three CpG sites in the *OXTR* gene moderated the association between experiences of physical abuse in childhood and symptoms of depression or anxiety in adulthood. Interestingly, the direction of these interactions varied between these CpG sites. For example, for the CpG site located within *OXTR* promoter region, participants with lower methylation levels reported higher depression and anxiety levels if they reported experiences of physical abuse. Taken together, these findings provide preliminary evidence for epigenome-environment interactions. Differences in DNAm levels may thus help explain why some adults are more likely to suffer from depressive symptoms following maltreatment experiences in childhood and adolescence, while others do not (or less so).

The present study aimed to extend current evidence suggesting that DNAm may play a role in the association between maltreatment experiences in childhood and depressive symptoms in emerging adulthood. Specifically, we tested the putative mediating and moderating roles of DNAm. First, we expected that individual differences in DNAm levels of nine genes involved in the regulation of stress, emotions and behaviors (*COMT*, *FKBP5*, *IL6*, *IL10*, *MAOA*, *NR3C1*, *OXTR*, *SLC6A3*, and *SLC6A4*) would partly mediate (or explain) the association between maltreatment experiences in childhood and depressive symptoms in early adulthood. Second, we expected that DNAm of these candidate genes would also moderate (or modulate) the strength and/or direction of the maltreatment-depression association, whereby the strength of this association is expected to be stronger in participants with a high-risk DNAm profile (either high or low DNAm levels depending on the targeted gene) than in participants with a low-risk DNAm profile (either high or low DNAm levels depending on the targeted gene). In addition, as each CpG site usually exert a small effects in the prediction of complex phenotypes, such as depressive symptoms, we expected that the cumulative effect of all significant individual CpG sites would yield stronger mediating and moderating effects than their individual effects [37].

## Materials and methods

### Participants

The sample includes 160 men aged from 18 to 35 years recruited from the general population (*M* = 24.06, *SD* = 3.70). Participants were selected from a larger study whose general objective was to investigate the mechanisms by which victimization experiences (e.g., maltreatment, bullying) in childhood and adolescence are linked to higher levels of aggression in emerging adulthood. This group was targeted for three reasons. First, the prevalence of aggressive behaviors is higher in males than in females [47] and as DNAm profiles appear to differ according to participants’ biological sex [48], only men were initially investigated. Second, emerging adulthood represents a transitional stage from dependency (i.e., adolescence) to autonomy (i.e., adulthood), a stressful period in which young adults are at heightened risk to develop mental health problems [49]. Third, the biological and psychological consequences of child maltreatment are rarely investigated in this age group [50]. Of the 160 participants, four were removed because they had previously participated to a standardized stress test, resulting in a final sample of 156 participants.

### Procedure

Participants were recruited for a study about early life experiences via advertisements displayed on public billboards and posted online on the Center for Studies on Human Stress’s website. In order to recruit enough participants with a history of child maltreatment, the advertisements insisted on memories of childhood and adolescence. Trained research assistants conducted a short phone interview with interested individuals, in which the questions regarding child maltreatment experiences were asked in the context of ‘when you were growing up’. Eligible participants were invited to a single laboratory session at the Center for Studies on Human Stress, which lasted about three hours and a half. The sampling strategy was blind in regards to depressive symptoms. Upon their arrival at the laboratory, participants were once again informed about the study procedures and provided written consent. All participants provided saliva samples for DNA extraction approximately two hours after they took part into a standardized stress test. Participants completed a questionnaire measuring depressive symptoms at the end of the visit. This study was approved by the Ethics Committee of the Research Center of the Montreal Mental Health University Institute (Project no. 2014–146, 2013–014). Data collection took place between July 2013 and December 2014.

### Measures

#### Child maltreatment

Maltreatment experiences in childhood and adolescence were assessed retrospectively using the Childhood Trauma Questionnaire–Short form (CTQ-SF) [51], which measures five types of traumatic experiences, including emotional abuse, physical abuse, sexual abuse, emotional neglect, and physical neglect. Participants rated the extent to which each of the 28 items (25 clinical items and 3 validity items) corresponded to past experiences on a five-point Likert scale ranging from 1 = “never true” to 5 = “very often true”, leading to total scores 5 to 25 on each subscale. The severity of maltreatment experiences was calculated by summing up all 25 items. In this sample, participants’ scores varied from 25 to 76 (*M* = 37.43, *SD* = 11.04). Of note, the scores of three participants were winsorized by setting them to the highest value within three standard deviations from the sample’s mean to avoid the disproportional weight of these extreme scores in subsequent analyses.

#### Depressive symptoms

Current depressive symptoms were assessed using the Beck Depression Inventory-II (BDI-II) [52]. Participants rated each of the 21 items according to their severity in the past two weeks using a four-point Likert scale ranging from 0 = “not present” to 3 = “severe”. The severity of depressive symptoms was calculated by summing up all 21 items. In this sample, participants’ scores varied from 0 to 44 (*M* = 10.48, *SD* = 8.79). The score of three participants were winsorized according to the aforementioned strategy.

#### DNA methylation

Saliva samples were collected between July 2013 and December 2014 using the Oragene^®^ Self-Collection Kit (DNA Genotek, Ontario, Canada). DNA extraction and DNAm analysis were performed by Génome Québec (Québec, Canada) in July 2015, less than two years after saliva collection. Because of the proprietary reagents present in these kits, saliva samples can be stored up to five years at room temperature without significant DNA degradation [53]. Genomic DNA was extracted manually using the QIAamp^®^ DNA Blood Mini Kit (QIAGEN, Hilden, Germany). Genomic DNA was then treated with bisulfite using the EZ-DNA Methylation Gold Kit (Zymo Research, California, United States) according to the manufacturer’s protocol. DNAm levels of 442 CpG sites previously investigated within nine candidate genes (*COMT*, *FKBP5*, *IL6*, *IL10*, *MAOA*, *NR3C1*, *OXTR*, *SLC6A3* and *SLC6A4*, see S1 Table for detailed locations) were quantified using the EpiTYPER MassARRAY technology (Agena Bioscience, California, United States). DNAm levels at each CpG site were calculated as the percentage of the surface area of the peak representing the methylated fragment by the total surface area of the peaks of both methylated and unmethylated fragments. Methylation values varied from 0% (unmethylated) to 100% (fully methylated). CpG sites with high mass or low mass as well as duplicates were removed (n = 116). CpG sites with greater than 20% missing values were removed (n = 6) as well as the CpG sites with greater than 80% of zeros (n = 14). Finally, CpG sites within the same gene that were positively and perfectly correlated (*r* = 1.00) have been grouped into a single variable, resulting in a final sample of 191 CpG sites.

#### Potential confounding variables

Information about the sociodemographic (e.g., age, civil status, education), health (e.g., height, weight, physical and/or mental health problems), medication intake (e.g., antidepressants, anxiolytics, antipsychotics), and lifestyle characteristics (e.g., tobacco, alcohol, and/or drug consumption) were enquired during the phone interview. The Body Mass Index (BMI) was calculated for each participant by dividing their body weight (in kilograms) by the square of their body height (in meters).

### Statistical analyses

All statistical analyses were performed using R 3.6.0 [54]. We first tested the bivariate associations between maltreatment experiences, depressive symptoms and DNAm levels using a series of linear regressions in which DNAm level at each CpG site was the dependent variable and maltreatment experiences or depressive symptoms were included in the model as an independent variable. We also examined these associations within specific genomic regions, where the CpG sites of the same gene that were correlated at *r* ≥ 0.50 and located within 500 base pairs of each other were grouped. The mean scores of the designated CpG sites were then used.

To examine the mediating role of DNAm in the association between maltreatment experiences and depressive symptoms, we performed a mediation analysis, using the *mediation* package in R [55]. This method uses information from two linear regression models: (1) DNAm as the dependent variable and child maltreatment as the independent variable and (2) depressive symptoms as the dependent variable and child maltreatment and DNAm as independent variables. The mediating effect is considered statistically significant when the lower and the upper bounds of the 95% confidence interval of the indirect effect do not include zero. Confidence intervals were estimated by using 1,000 Monte Carlo simulations. To avoid unnecessary tests and thus minimize the likelihood of identifying false positives, the mediation analyses were only conducted for CpG sites with significant associations with both maltreatment experiences and depressive symptoms. The methylation levels of these CpG sites were then summed up to derive a cumulative index of methylation and included as a potential mediator in the linear regression model.

To examine the moderating role of DNAm, we performed a moderation analysis, using the *interactions* package. This method uses information from a linear regression model: child maltreatment, DNAm and their interaction term as independent variables and depressive symptoms as the independent variable. All independent variables were mean-centered prior to analyses. The moderating effect was considered statistically significant when the estimate of the interaction term between child maltreatment and DNAm had an observed *p* < 0.05. Significant interactions were decomposed and illustrated by using the simple slopes analysis, which depicts the association between child maltreatment and depressive symptoms at one standard deviation above and below the mean methylation level of the identified CpG sites. The methylation levels of all CpG sites that modified the strength of the association between maltreatment experiences and depressive symptoms were grouped into tertiles and given a score of -1, 0 or 1 depending on the direction of associations. These scores were then summed up to derive a cumulative index of methylation and included as a potential moderator in the linear regression model.

Significant associations were rerun with confounding variables for which a unique association of *p* < 0.10 was detected between child maltreatment, DNAm or depressive symptoms. To do so, we tested, in preliminary analyses, the associations between these three variables and a wide range of individual characteristics known to covary with child maltreatment and depressive symptoms or affecting DNAm profiles, including age, smoking, drug consumption, alcohol consumption, and BMI. Only age and drug consumption were associated with the methylation level of several CpG sites as well as with depressive symptoms. These variables were then controlled for in the aforementioned linear models, mediation models and moderation models.

Correction for multiple testing was performed using the false discovery rate (FDR). As none of the results were significant after this correction at FDR < 0.20 and given the exploratory nature of this study, all results nominally significant at *p* < 0.05 were deemed of interest and discussed accordingly.

## Results

### Association between child maltreatment and depressive symptoms

Child maltreatment was significantly associated with depressive symptoms (*B* = 0.25, *p* < 0.001), whereby child maltreatment explained 10% of the variance of depressive symptoms (*R*^*2*^ = 0.10, *F*(1, 154) = 17.39, *p* = < 0.001).

### Associations between child maltreatment and DNA methylation

Child maltreatment was significantly associated with the methylation levels of 9 CpG sites, namely 1 site in the *MAOA* gene, 2 sites in the *NR3C1* gene, 4 sites in the *SLC6A3* gene, and 2 sites in the *SLC6A4* gene (see Unadjusted Models in Table 1). Estimates of explained variance varied between 2.51% to 4.82%. After adjusting for age and drug consumption, only 8 CpG sites remained significant (see Adjusted Models in Table 1). None of these associations survived multiple testing correction. In addition, grouping the CpG sites per regions did not yield additional significant findings.

**Table 1:** Significant associations between child maltreatment and DNA methylation.

CpG name	Position	DNA methylation							
		Unadjusted Models				Adjusted Models			
		*B*	*SE*	*p*	*R* ^*2*^	*B*	*SE*	*p*	*R* ^*2*^
***MAOA***									
MAOA_2_CpG_12and13	chrX:43515458 chrX:43515468	-0.029	0.015	0.048	0.025	-0.026	0.015	0.074	0.052
***NR3C1***									
NR3C1_1_CpG_9	chr5:142784413	0.030	0.013	0.021	0.034	0.029	0.013	0.022	0.065
NR3C1_2_CpG_49to52	chr5:142783299 chr5:142783303 chr5:142783310 chr5:142783314	0.041	0.015	0.006	0.048	0.040	0.015	0.008	0.052
***SLC6A3***									
SLC6A3_1_CpG_4	chr5:1446537	0.079	0.031	0.012	0.040	0.080	0.032	0.012	0.041
SLC6A3_1_CpG_8to11	chr5:1446488 chr5:1446485 chr5:1446478 chr5:1446474	-0.022	0.010	0.031	0.030	-0.024	0.010	0.016	0.068
SLC6A3_1_CpG_16	chr5:1446430	-0.050	0.021	0.018	0.036	-0.053	0.021	0.015	0.043
SLC6A3_2_CpG_2to4	chr5:1446371 chr5:1446369 chr5:1446367	0.026	0.011	0.019	0.035	0.027	0.011	0.016	0.043
***SLC6A4***									
SLC6A4_2_CpG_28and29	chr17:28562786 chr17:28562783	-0.010	0.005	0.034	0.029	-0.010	0.005	0.038	0.066
SLC6A4_3_CpG_36	chr17:28562435	-0.029	0.015	0.046	0.026	-0.032	0.015	0.027	0.060

*Note*. Based on GRCh37/hg19 coordinates.

### Associations between DNA methylation and depressive symptoms

Depressive symptoms were significantly associated with the methylation levels of 14 CpG sites, namely, 1 site in the *IL6* gene, 1 site in the *IL10* gene, 2 sites in the *NR3C1* gene, 1 site in the *OXTR* gene, 3 sites in the *SLC6A3* gene, and 6 sites in the *SLC6A4* gene (see Unadjusted Models in Table 2). Estimates of explained variance varied between 2.54% to 4.96%. Of these, 3 CpG sites (1 site in the *NR3C1* gene, 1 site in *SLC6A3* gene, and 1 site *SLC6A4* gene) were also associated with child maltreatment. After adjusting for age and drug consumption, only 8 CpG sites remained significant, including the 2 of the 3 sites also associated with child maltreatment (see Adjusted Models in Table 2). None of these associations survived multiple testing correction. Once again, grouping the CpG sites per regions did not yield additional significant findings.

**Table 2:** Significant associations between depressive symptoms and DNA methylation.

CpG names	Position	DNA methylation							
		Unadjusted Models				Adjusted Models			
		*B*	*SE*	*p*	*R* ^*2*^	*B*	*SE*	*p*	*R* ^*2*^
***IL6***									
IL6_2_CpG_3and4	chr7:22764029 chr7:22764031	-0.082	0.037	0.028	0.033	-0.093	0.384	0.015	0.046
***IL10***									
IL10_2_CpG_4	chr1:206940003	0.110	0.053	0.040	0.027	0.084	0.054	0.119	0.082
***NR3C1***									
NR3C1_2_CpG_49to52	chr5:142783299 chr5:142783303 chr5:142783310 chr5:142783314	0.043	0.019	0.027	0.031	0.048	0.020	0.016	0.044
NR3C1_2_CpG_61to63	chr5:142783380 chr5:142783384 chr5:142783386	0.099	0.039	0.013	0.039	0.107	0.041	0.009	0.048
***OXTR***									
OXTR_2_CpG_11	chr3:8810699	-0.046	0.021	0.032	0.029	-0.035	0.022	0.107	0.058
***SLC6A3***									
SLC6A3_1_CpG_7	chr5:1446498	-0.033	0.016	0.041	0.027	-0.035	0.016	0.033	0.044
SLC6A3_1_CpG_12	chr5:1446462	-0.061	0.029	0.035	0.028	-0.054	0.029	0.064	0.055
SLC6A3_1_CpG_16	chr5:1446430	-0.065	0.027	0.018	0.036	-0.064	0.028	0.023	0.038
***SLC6A4***									
SLC6A4_2_CpG_26	chr17:28562826	-0.014	0.006	0.033	0.029	-0.009	0.006	0.154	0.090
SLC6A4_2_CpG_28and29	chr17:28562786 chr17:28562783	-0.013	0.006	0.024	0.033	-0.011	0.006	0.069	0.060
SLC6A4_3_CpG_1and2	chr17:28562751 chr17:28562749	-0.021	0.010	0.048	0.025	-0.022	0.011	0.039	0.031
SLC6A4_3_CpG_9to12	chr17:28562706 chr17:28562703 chr17:28562700 chr17:28562691	-0.050	0.018	0.005	0.050	-0.042	0.018	0.020	0.111
SLC6A4_3_CpG_22	chr17:28562596	-0.066	0.029	0.022	0.034	-0.065	0.030	0.030	0.038
SLC6A4_3_CpG31to33	chr17:28562499 chr17:28562492 chr17:28562489	-0.041	0.018	0.021	0.034	-0.034	0.018	0.058	0.061

*Note*. Based on GRCh37/hg19 coordinates.

### Mediation analyses

**Fig 1:**
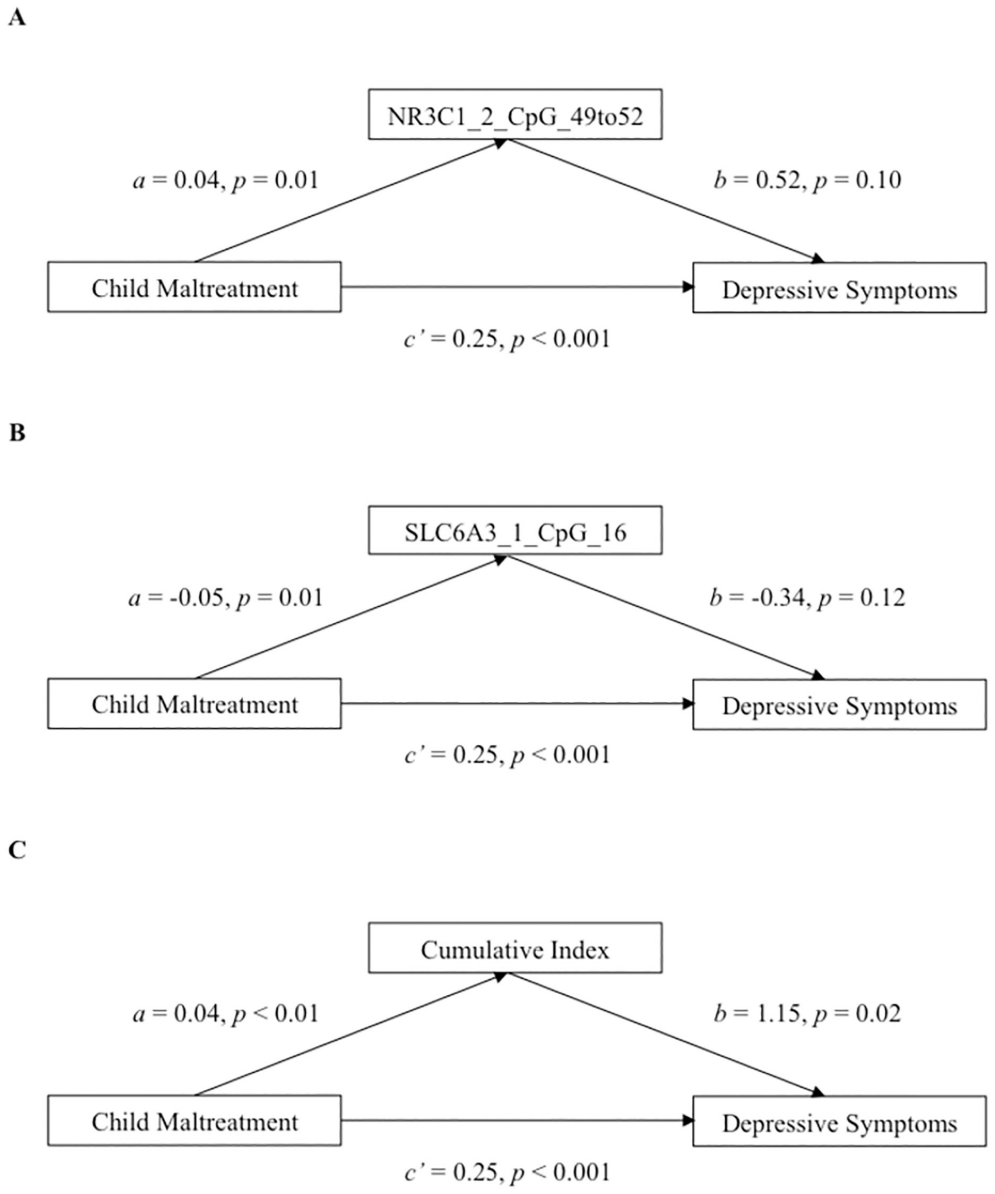
Mediation analyses. (A) Adjusted model for NR3C1_2_CpG_49to52. (B) Adjusted model for SLC6A3_1_CpG_16. (C) Adjusted model for the index of cumulative methylation.

The mediation analyses focused on the CpG sites associated with both child maltreatment and depressive symptoms after controlling for age and drug consumption. The methylation level of NR3C1_2_CpG_49to52 did not mediate the association between maltreatment and depressive symptoms (Fig 1A). While the association between maltreatment experiences and the methylation level of NR3C1_2_CpG_49to52 was still significant (*B* = 0.04, *p* = 0.01), the association between the methylation level of NR3C1_2_CpG_49to52 and depressive symptoms was no longer significant when controlling for maltreatment experiences (*B* = 0.47, *p* = 0.15). The bootstrapped unstandardized coefficient representing the indirect effect was 0.02, for which the 95% confidence interval ranged from -0.006 to 0.050, indicating a non-significant indirect effect (*p* = 0.15). After adjusting for age and drug consumption, the bootstrapped unstandardized indirect effect was practically unchanged (*B* = 0.02, 95% CI = -0.003 to 0.060, *p* = 0.13).

The methylation level of SLC6A3_1_CpG_16 did not either mediate the association between child maltreatment and depressive symptoms (Fig 1B). Once more, while the association between maltreatment experiences and the methylation level of SLC6A3_1_CpG_16 was significant (*B* = -0.05, *p* = 0.02), the association between the methylation level of SLC6A3_1_CpG_16 and depressive symptoms was no longer significant when controlling for maltreatment experiences (*B* = -0.39, *p* = 0.08). The bootstrapped unstandardized indirect effect was 0.02, and the 95% confidence interval ranged from -0.003 to 0.060, suggesting a non-significant indirect effect (*p* = 0.11). After adjusting for age and drug consumption, the bootstrapped unstandardized indirect effect was practically unchanged (*B* = 0.02, 95% CI = -0.003 to 0.050, *p* = 0.11).

Interestingly, these 2 CpG sites cumulatively mediated the association between maltreatment experiences and depressive symptoms, and this, even after controlling for age and drug consumption (Fig 1C). The bootstrapped unstandardized coefficient of the indirect effect was 0.04, with 95% confidence intervals ranged from 0.008 to 0.091 indicating a significant indirect effect. That is, maltreatment experiences were associated with higher levels of the index of cumulative methylation (*B* = 0.04, *p* < 0.001), which was, in turn, associated to higher depressive symptoms (*B* = 1.12, *p* = 0.02). Nevertheless, after accounting for the indirect effect of the index of cumulative methylation, maltreatment experiences were still significantly associated with depressive symptoms (*B* = 0.25, *p* < 0.001), suggesting a partial mediation. The index of cumulative methylation mediated approximately 16% of the association between maltreatment experiences and depressive symptoms (*B* = 0.16, 95% CI = 0.03 to 0.41, *p* = 0.02).

### Moderation analyses

A total of 12 CpG sites significantly moderated the association between maltreatment experiences and depressive symptoms, including 1 site in the *FKBP5* gene, 3 sites in the *MAOA* gene, 3 sites in the *NR3C1* gene, 2 sites in the *SLC6A3* gene, and 3 sites in the *SLC6A4* gene (see Unadjusted Models in Table 3). Estimates of explained variance varied between 12.4% and 18.4%. Once age and drug consumption were included in the models, the methylation levels of 14 CpG sites significantly moderated the association between maltreatment experiences and depressive symptoms, namely 2 sites in the *FKBP5* gene, 2 sites in the *IL10* gene, 1 site in the *MAOA* gene, 3 sites in the *NR3C1* gene, 3 sites in the *SLC6A3* gene, and 3 sites in the *SLC6A4* gene (see Adjusted Models in Table 3). The interactions between maltreatment experiences and the methylation levels of each of these 14 CpG sites are depicted in S1 Fig. However, none of these interactions survived multiple testing correction. Grouping the CpG sites per regions did not yield additional significant findings.

**Table 3:** Significant interactions between child maltreatment and DNA methylation predicting depressive symptoms.

CpG names	Position	DNA methylation							
		Unadjusted Models				Adjusted Models			
		*B*	*SE*	*p*	*R* ^*2*^	*B*	*SE*	*p*	*R* ^*2*^
***FKBP5***									
FKBP5_1_CpG_3	chr6:35558489	0.022	0.011	0.047	0.126	0.022	0.011	0.043	0.182
FKBP5_1_CpG_4	chr6:35558514	0.020	0.011	0.071	0.124	0.024	0.011	0.026	0.187
***IL10***									
IL10_2_CpG_5	chr1:206939984	-0.034	0.019	0.075	0.124	-0.043	0.019	0.022	0.189
IL10_2_CpG_6	chr1:206939954	-0.025	0.014	0.066	0.137	-0.032	0.014	0.021	0.195
***MAOA***									
MAOA_2_CpG_7to9	chrX:43515403 chrX:43515413 chrX:43515419	-0.075	0.035	0.035	0.131	-0.056	0.035	0.115	0.179
MAOA_2_CpG_27	chrX:43515647	-0.022	0.009	0.016	0.168	-0.015	0.009	0.101	0.214
MAOA_3_CpG_1	chrX:43515676	0.025	0.012	0.033	0.184	0.025	0.011	0.031	0.245
***NR3C1***									
NR3C1_1_CpG_5	chr5:142784370	-0.096	0.050	0.055	0.124	-0.098	0.048	0.045	0.184
NR3C1_2_CpG_23and24	chr5:142783121 chr5:142783129	-0.095	0.047	0.043	0.127	-0.085	0.046	0.067	0.179
NR3C1_2_CpG_60	chr5:142783361	-0.200	0.096	0.039	0.136	-0.191	0.094	0.044	0.189
NR3C1_3_CpG_8	chr5:142782723	0.265	0.012	0.024	0.137	0.270	0.113	0.019	0.194
***SLC6A3***									
SLC6A3_1_CpG_7	chr5:1446498	-0.072	0.042	0.086	0.135	-0.085	0.041	0.037	0.199
SLC6A3_2_CpG_28to30	chr5:1446121 chr5:1446119 chr5:1446113	-0.081	0.037	0.029	0.130	-0.083	0.036	0.023	0.188
SLC6A3_2_CpG_35to37	chr5:1446079 chr5:1446076 chr5:1446068	-0.089	0.038	0.021	0.135	-0.097	0.037	0.010	0.196
***SLC6A4***									
SLC6A4_3_CpG_13and14	chr17:28562685 chr17:28562683	0.082	0.038	0.036	0.129	0.079	0.037	0.036	0.186
SLC6A4_3_CpG_23and24	chr17:28562572 chr17:28562567	0.069	0.034	0.042	0.126	0.070	0.033	0.035	0.186
SLC6A4_3_CpG_36	chr17:28562435	0.084	0.033	0.013	0.140	0.085	0.032	0.009	0.197

*Note*. Based on GRCh37/hg19 coordinates. Estimates *(B)* represent the coefficient of the interaction between maltreatment experiences and DNAm levels.

**Fig 2:**
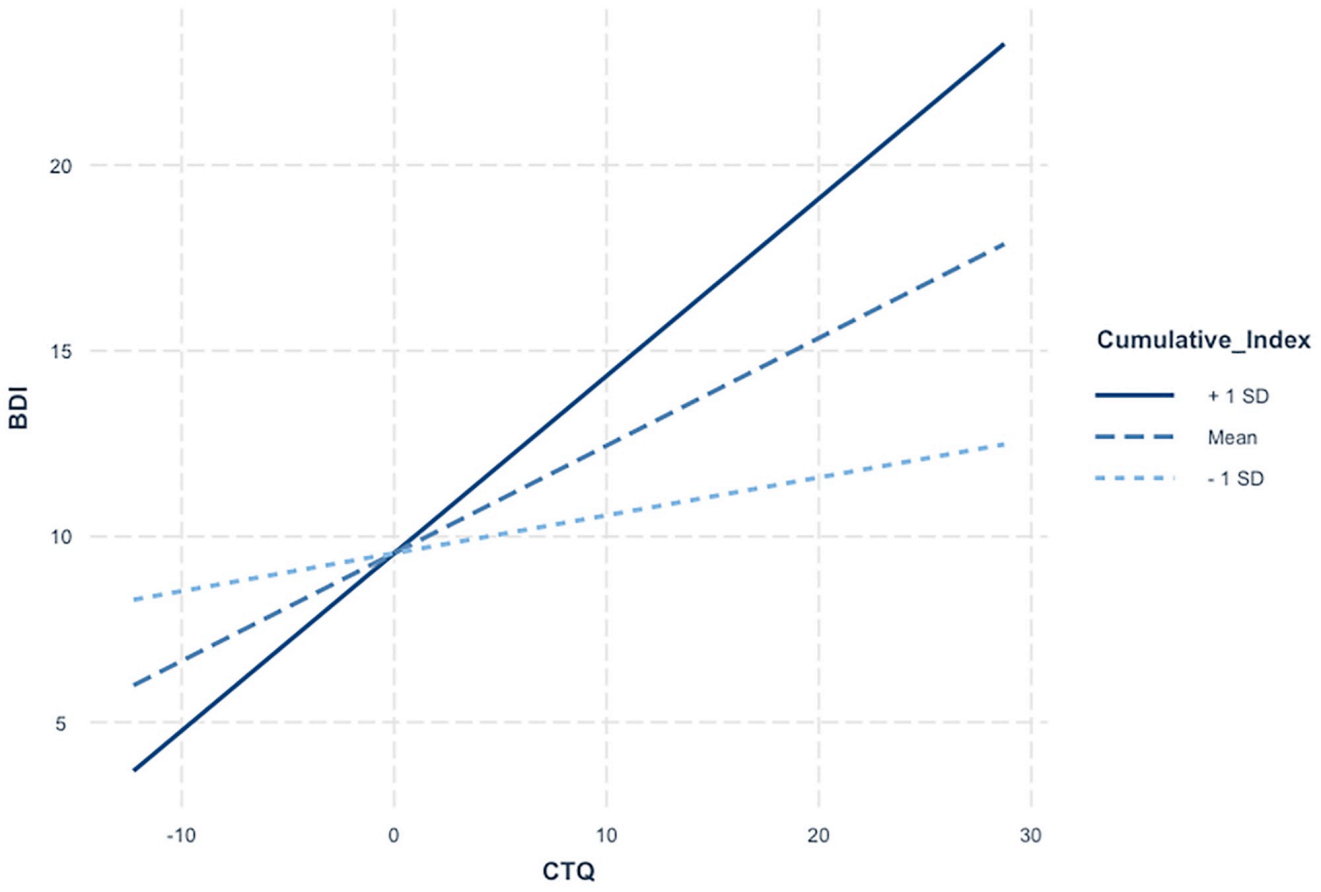
A visual representation of the association between child maltreatment and depressive symptoms according to a cumulative index of methylation. Fig 2 depicts the conditional association between maltreatment experiences and depressive symptoms according to increasing DNAm levels, depicted in three equal groups.

Interestingly, these 14 CpG sites cumulatively moderated the association between maltreatment experiences and depressive symptoms (*B* = 0.04, *p* < 0.01), while still controlling for age and drug consumption. Specifically, stronger associations between maltreatment experiences and depressive symptoms were noted as the cumulative index of methylation, derived from the methylation levels of the 14 CpG sites previously identified to independently moderate this association, increased. While no association was detected for participants with lower scores in this cumulative index (*B* = 0.12, *p* = 0.11), significant effects emerged for those who had moderate (*B* = 0.30, *p* < 0.01) to higher scores (*B* = 0.49, < 0.01; see Fig 2). This interactive model explained 28% of the variance of depressive symptoms (*R*^*2*^ = 0.28, *F*(5, 136) = 10.35, *p* = < 0.001).

## Discussion

In the present study, we aimed to extend preliminary reports suggesting that DNAm may play a role in the association between maltreatment experiences in childhood and depressive symptoms in emerging adulthood. Specifically, we examined whether this association could be explained, in part, by the DNAm levels of nine candidate genes involved in the regulation of stress, emotions and behaviors (i.e., mediation) and whether the strength and/or direction of this association could be modulated by individual differences in DNAm levels (i.e., moderation). Consistent with a previous study on this sample [56], we observed a positive association between maltreatment experiences and depressive symptoms. Four observations are worth highlighting.

First, we found that maltreatment experiences were associated with DNAm levels of eight CpG sites (out of 191). In line with previous studies [57, 58], the effect sizes of the uncovered associations between child maltreatment and salivary DNAm were relatively small (*B* < 8%). Unfortunately, none of the CpG sites deemed nominally significant (*p* < 0.05) survived multiple testing correction. Nonetheless, these signals remain of interest for future investigations and contribute to foster a greater collective knowledge about the expected role of DNAm in the association between maltreatment experiences and depressive symptoms. Specifically, our results indicate that adults who reported higher levels of maltreatment experiences exhibited higher levels of methylation at two CpG sites across *NR3C1* promoter region (out of 34), including one CpG site within the exon 1_B_ and one CpG site within the exon 1_C_. Labonté et al. [23] previously found that men who died by suicide and who had a history of child abuse showed differential methylation levels in *NR3C1* promoter region in the hippocampus, namely two CpG sites within the exon 1_C_ (out of 18) and three CpG sites within the exon 1_H_ (out of 13) compared to men who died by suicide without such a history. Contrary to our findings, Labonté et al. [23] did not observed differential methylation levels at any of the 12 CpG sites investigated within the exon 1_B_. In addition, the two CpG sites that we found to be associated with maltreatment experiences in our study did not correspond to those reported by Labonté et al. [23] Although the majority of studies found a positive association between child maltreatment and *NR3C1* methylation levels [13], inconsistencies exist, especially when focusing on specific (individual) CpG sites. These inconsistencies may be partly due to the use of different biological tissues to measure DNAm. Indeed, as DNAm plays an important role in cell differentiation [59], it is unclear whether the observed inter-individual differences in DNAm levels are attributable to the exposure to maltreatment experiences or tissue specificity. Our results also indicate that adults who reported higher levels of maltreatment experiences exhibited lower levels of methylation at two CpG sites across *SLC6A4* promoter region (out of 31). Our findings, however, contrast with the majority of previous studies, who rather reported higher levels of *SLC6A4* methylation in blood samples of adults exposed to maltreatment experiences in childhood [13, 14]. It is important to highlight that these studies mainly focused on experiences of sexual abuse in samples primarily composed of female participants. As women tend to report more experiences of sexual abuse than men [60], we pondered that these associations are likely to be sexually dimorphic. Interestingly, our results indicate that adults who reported higher levels of maltreatment experiences exhibited higher levels of methylation at two CpG sites and lower levels of methylation at two CpG sites, all four located within *SLC6A3* promoter region (out of 27). To the best of our knowledge, no study has yet investigated the association between maltreatment experiences and *SLC6A3* methylation levels in humans. Although some studies reported differential methylation levels of the *FKBP5*, *IL6* and *MAOA* genes between adults with and without a history of child maltreatment [13, 14], we did not find evidence for associations between maltreatment experiences and methylation levels of any CpG sites within those genes. Our findings are thus in line with other studies that also reported no association between maltreatment experiences and the *FKBP5* methylation levels [26, 32, 61, 62] or *MAOA* methylation levels [37]. In addition to variation related to biological tissues used to measure DNAm, we speculated that the strategy to measure and operationalize child maltreatment (i.e., dichotomous variable or continuous variable) might further exacerbate these inconsistencies. As the majority of previous studies reported no overall association between maltreatment experiences and *OXTR* methylation levels [13, 14], we also found no association between maltreatment experiences and *OXTR* methylation levels at any of the CpG sites investigated within this gene. Nonetheless, additional studies are needed to further test the presence, strength and direction of the association between maltreatment experiences and DNAm levels.

Second, we found that current depressive symptoms were associated with DNAm levels of eight CpG sites (out of 191). Here again, the observed effect sizes were relatively small (*B* < 12%) and none of the CpG sites deemed nominally significant (*p* < 0.05) survived multiple testing correction. Specifically, adults who reported higher levels of depressive symptoms exhibited higher levels of methylation at two CpG sites across the *NR3C1* promoter region (out of 34). Our findings are in line with some studies that also detected higher levels of *NR3C1* methylation in blood samples of depressed adults in comparison to controls [26, 37, 63]. To the best of our knowledge, we are the first to investigate the association between depressive symptoms and *NR3C1* methylation levels in saliva samples of adults. Thus, it is quite noteworthy that our findings are similar to those examining *NR3C1* methylation in blood samples. Our results also indicate that adults who reported higher levels of depressive symptoms exhibited lower levels of methylation at three CpG sites across *SLC6A4* promoter region (out of 31). Although the majority of studies found higher *SLC6A4* methylation levels, some reported lower *SLC6A4* methylation levels [38]. In addition to the absence of a “real” effect, inconsistent findings may be partly explained by the use of different biological tissues to extract DNA or the composition of the sample (population-based vs. clinically-based samples). To the best of our knowledge, even if dopamine plays a role in motivation, mood and cognition [64], no candidate gene study has yet investigated the association between depressive symptoms and *SLC6A3* methylation levels. Interestingly, our results indicate that adults who reported higher levels of depressive symptoms exhibited lower levels of methylation at two CpG sites across the *SLC6A3* promoter region (out of 27). Our results also indicate that adults who reported higher levels of depressive symptoms exhibited lower levels of methylation at one CpG site across *IL6* promoter region (out of 10), which is consistent with the study of Ryan et al. [65], who also reported lower levels of methylation at one CpG site across *IL6* promoter region (out of 4) in saliva samples of depressed adults. Although some studies reported differential methylation levels CpG sites in the *FKBP5*, *MAOA* and *OXTR* genes between depressed and healthy participants [38], we did not find evidence for associations between maltreatment experiences and methylation levels of any CpG sites within those genes. Similarly to the associations between maltreatment experiences and DNAm levels, additional studies with large prospective cohorts research design are needed to test these associations more robustly.

Third, we found that the methylation levels of two CpG sites, for which bivariate associations with maltreatment experiences and depressive symptoms were nominally significant (*p* < 0.05), did not independently explain (or mediate) the association between maltreatment experiences and depressive symptoms. To the best of our knowledge, only four studies have tested the putative mediating role of DNAm in the maltreatment-depression association. We were not able to replicate the findings reported for the *NR3C1* and *MAOA* genes, for which DNAm levels were found to explain the maltreatment-depression association [36, 37]. Nonetheless, our results somewhat echo with the other two studies that targeted other candidate genes, such as *FKBP5* [32] and *OXTR* [43], for which DNAm levels did not explain the maltreatment-depression association. Interestingly, we found that a cumulative index of methylation, derived from the methylation levels of the two aforementioned CpG sites, explained 16% of the association between maltreatment experiences in childhood and depressive symptoms in adulthood. Our results somewhat echo with the study of Peng et al. [37], which highlighted the importance of testing the cumulative epigenetic effect of multiple CpG sites on complex phenotypes, which may help to unravel the molecular mechanisms through which early-life stress may become biologically embedded in stress-related neurobiological systems supporting mental health. Although additional studies with larger samples are required to replicate these preliminary findings, several alternative explanations deserve further consideration. First, DNAm may serve as a biological marker (i.e., biomarker) signalling exposure to child maltreatment and/or the presence of a depressive symptomatology, which could be of clinical utility, even without being directly involved in the causal pathways leading up to psychopathology. For instance, DNAm profiles have already been shown to be useful in the detection and prognosis of cancer as well as the prediction of response to treatment [3]. Second, it is also possible that child maltreatment indeed influences DNAm patterns of stress-related genes in the days, weeks and years following these experiences, but that they represent only a small fraction of all the neurobiological changes leading to psychopathology. The multifactorial and dynamic nature of these pathways certainly complicates the detection of significant indirect effects.

Fourth, we found that the methylation levels of fourteen CpG sites (out of 191) independently modified (or moderated) the strength of the association between maltreatment experiences and depressive symptoms and this, in a nominally significant fashion (*p* < 0.05). On the one hand, our results indicated that child maltreatment was associated with higher risk of depressive symptoms among young male adults with moderate to high *FKBP5* methylation levels at two CpG sites, but not for those with low *FKBP5* methylation levels at these two CpG sites. A similar direction pattern was also observed for the methylation levels of one CpG site within the *MAOA* gene, one CpG site within the *NR3C1* gene and three CpG sites within the *SLC6A4* gene. On the other hand, our results indicate that child maltreatment was associated with higher risk of depressive symptoms among young male adults with low to moderate *IL10* methylation levels at two CpG sites, but not for those with high *IL10* methylation levels at these two CpG sites. A similar direction pattern was also observed for two CpG sites within the *NR3C1* gene and three CpG sites within the *SLC6A3* gene. Although we were able to replicate the findings reported by Radkte et al. [28], for which *NR3C1* methylation levels were found to interact with child maltreatment to predict psychopathology, we were not able to replicate the findings reported by Smearman et al. [43], for which *OXTR* methylation levels were found to interact with physical abuse to predict depression and anxiety. Interestingly, we found that a cumulative index of methylation, derived from the methylation levels of the fourteen aforementioned CpG sites, significantly modulated the strength of the association between maltreatment experiences in childhood and depressive symptoms in adulthood, whereby young male adults who exhibited higher methylation risk scores reported higher levels of depressive symptoms following maltreatment experiences in childhood. Specifically, child maltreatment was associated with higher levels of depressive symptoms among young male adults with a moderate to high risk DNAm profile, but not for those with a low risk DNAm profile. Taken together, these independent and cumulative signals offer additional support to the moderating role of DNAm in the association between maltreatment experiences and depressive symptoms, although existing theoretical frameworks rather advance its mediating role.

Our findings should be considered in light of several limitations. First, we were unable to control for cell heterogeneity within our study. Since different types of cells show distinct patterns of methylation [59], it may have influenced DNAm profiles, which may have contributed to our reported null findings. Second, the sample size used in this study is small (*N* = 156), which may explain why we were not able to replicate some of the previous findings and that none of our results survived multiple testing correction. We thus recommend caution in interpreting these preliminary results, especially at the CpG level. Third, our sample only included young adult male participants. Therefore, our results may not apply to female participants or to younger or older populations, as DNAm varies in function of age and sex [48, 66]. Fourth, our participants were recruited from the general population, so that only 4.5% of the sample reported severe levels of depressive symptoms. Therefore, our results may not apply to clinical populations. Moreover, only 35.9% of the sample reported at least one type of maltreatment experiences. The restricted variance in these key variables may have further undermined our statistical power to detect the expected associations. Fifth, we used a self-reported and retrospective questionnaire to assess child maltreatment, which may be subject to recall biases. However, previous studies reported that memories of maltreatment experiences in childhood and adolescence appear to be reliable in adulthood [67]. Moreover, recall bias seems to explain less than 1% of the variance of child maltreatment scores [68]. Sixth, we cannot determine the temporal sequence of events since this study is cross-sectional. We also did not have the statistical power to investigate whether the association between maltreatment and DNAm varied according to individual characteristics (e.g., sex, genetic background) and/or maltreatment characteristics (e.g., onset, recency). For example, using a prospective design, Cicchetti et al. [69] not only found genome-wide differences in DNAm levels between maltreated and non-maltreated children, but also significant interactions between maltreatment, its onset and sex on DNAm levels of specific genes (e.g., *ALDH2*). Seventh, the study relied on data analyzed in 2015 according to a candidate gene approach. As candidate gene and epigenome-wide studies share little to no overlap between target sequences (e.g., [30]), candidate gene studies remain relevant to examine the role of specific genes in the association between maltreatment experiences and depressive symptoms, as well as to inform future meta-analytic work targeting these genes.

Despite these limitations, our findings extend previous research that alluded to or directly tested the putative mediating or moderating roles of DNAm in the association between child maltreatment and depressive symptoms in emerging adulthood. Our study provides preliminary support for the cumulative effect of DNAm levels detected within several candidate genes on the emergence or aggravation of depressive symptoms following maltreatment experiences. The relative value of using methylation risk scores to examine the cumulative effect of DNAm levels of multiple CpG sites spanning multiple genes should be further investigated, as well as complemented by using other methylation risk scores, such as the EpiStress score [70], the DunedinPACE [71] and epigenetic clocks [72]. Our findings are also consistent with the added value of using measures of maltreatment that span a wider range of severity and types of exposure [13]. Nevertheless, the putative effects of child maltreatment on DNAm should also be considered along other family risk factors (e.g., maternal and/or paternal psychopathology, alcohol and/or drug abuse), as preliminary evidence suggests that these additional stressful family experiences may have a cumulative effect on DNAm levels above and beyond child maltreatment (e.g., [73]). Moreover, the role of DNAm could be conditional on the allelic variations within the selected candidate genes (e.g., [74]). Finally, our study provides additional, albeit partial, support to the possibility that DNAm may further help to understand how or for whom child maltreatment may increase risk for depression later in life. Future studies would benefit from the collection of prospective measures of child maltreatment, DNAm levels and depressive symptoms, as well as the functional characterisation of DNAm findings, such as gene expression, to enhance understanding of the role of DNAm in the emergence or aggravation of depressive symptoms following maltreatment experiences.

## Supporting information

S1 Table Candidate genes. Based on GRCh37/hg19 coordinates.
(DOCX)

S2 Table Associations between child maltreatment and DNA methylation. Based on GRCh37/hg19 coordinates.
(DOCX)

S3 Table Associations between depressive symptoms and DNA methylation. Based on GRCh37/hg19 coordinates.
(DOCX)

S1 Fig Moderation analyses. (A) Adjusted model for FKBP5_1_CpG_3. (B) Adjusted model for FKBP5_1_CpG_4. (C) Adjusted model for IL10_2_CpG_5. (D) Adjusted model for IL10_2_CpG_6. (E) Adjusted model for MAOA_3_CpG_1. (F) Adjusted model for NR3C1_1_CpG_5.
(TIF)

S2 Fig Moderation analyses. (G) Adjusted model for NR3C1_2_CpG_60. (H) Adjusted model for NR3C1_3_CpG_8. (I) Adjusted model for SLC6A3_1_CpG_7. (J) Adjusted model for SLC6A3_2_CpG_28to30. (K) Adjusted model for SLC6A3_2_CpG_35to37. (L) Adjusted model for SLC6A4_3_CpG_13and14.
(TIF)

S3 Fig Moderation analyses. (M) Adjusted model for SLC6A4_3_CpG_23and24. (N) Adjusted model for SLC6A4_3_CpG_36.
(TIF)
