# Supplementary material for: Association between child maltreatment and depressive symptoms in emerging adulthood: The mediating and moderating roles of DNA methylation
Source: PLoS One. 2023 Jan 12;18(1):e0280203. doi: 10.1371/journal.pone.0280203 (PMC9836296; doi:10.1371/journal.pone.0280203)
Supplement: S1 Table — Based on GRCh37/hg19 coordinates. (DOCX) [file pone.0280203.s001.docx]

| **S1 Table. Candidate Genes.** | | | |
| --- | --- | --- | --- |
| Gene | Fragment | Coordinates | CpG Sites |
|  |  |  |  |
| *COMT* | 1 | chr22:19950027-19950348 | 16 |
|  | 2 | chr22:19929067-19929331 | 37 |
|  |  |  |  |
| *FKBP5* | 1 | chr6:35558387-35558567 | 5 |
|  |  |  |  |
| *IL6* | 1 | chr7:22763499-22763846 | 11 |
|  | 2 | chr7:22763911-22764031 | 4 |
|  |  |  |  |
| *IL10* | 1 | chr1:206940522-206940.11 | 6 |
|  | 2 | chr1:206840215-206939313 | 9 |
|  |  |  |  |
| *MAOA* | 1 | chrX:43514936-43515089 | 8 |
|  | 2 | chrX:43515295-43515647 | 27 |
|  | 3 | chrX:43515676-43515991 | 6 |
|  |  |  |  |
| *NR3C1* | 1 | chr5:142784279-142784593 | 15 |
|  | 2 | chr5:142782988-142783500 | 84 |
|  | 3 | chr5:142782766-142782551 | 26 |
|  |  |  |  |
| *OXTR* | 1 | chr3:8809307-8809564 | 26 |
|  | 2 | chr3:8810889-8810647 | 14 |
|  |  |  |  |
| *SLC6A3* | 1 | chr5:104460585-1446430 | 16 |
|  | 2 | chr5:1446393-1446001 | 45 |
|  |  |  |  |
| *SLC6A4* | 1 | chr17:28563424-28563054 | 17 |
|  | 2 | chr17:28563020-28562783 | 29 |
|  | 3 | chr17:28562751-28562388 | 41 |

*Note.* Based on GRCh37/hg19 coordinates.
