# Supplementary material for: Association between child maltreatment and depressive symptoms in emerging adulthood: The mediating and moderating roles of DNA methylation
Source: PLoS One. 2023 Jan 12;18(1):e0280203. doi: 10.1371/journal.pone.0280203 (PMC9836296; doi:10.1371/journal.pone.0280203)
Supplement: S2 Table — Based on GRCh37/hg19 coordinates. (DOCX) [file pone.0280203.s002.docx]

| **S2 Table. Associations between child maltreatment and DNA methylation.** | | | | | | | | | | | |
| --- | --- | --- | --- | --- | --- | --- | --- | --- | --- | --- | --- |
| CpG Name | Position | DNA Methylation | | | | | | | | | |
|  |  | Unadjusted Models | | | | | Adjusted Models | | | | |
|  |  | *B* | *SE* | *p* | *R2* | *FDR* | *B* | *SE* | *p* | *R2* | *FDR* |
| ***COMT*** |  |  |  |  |  |  |  |  |  |  |  |
| COMT_1_CpG_3 | chr22:19950055 | -0.042 | 0.063 | 0.501 | 0.003 | 0.912 | -0.056 | 0.062 | 0.374 | 0.042 | 0.878 |
| COMT_1_CpG_4 | chr22:19950064 | 0.018 | 0.075 | 0.816 | 0.000 | 0.982 | -0.003 | 0.073 | 0.967 | 0.076 | 0.994 |
| COMT_1_CpG_5 | chr22:19950158 | -0.031 | 0.079 | 0.691 | 0.001 | 0.982 | -0.051 | 0.077 | 0.509 | 0.075 | 0.893 |
| COMT_1_CpG_7 | chr22:19950222 | -0.096 | 0.123 | 0.433 | 0.004 | 0.909 | -0.124 | 0.122 | 0.311 | 0.043 | 0.878 |
| COMT_1_CpG_8 | chr22:19950236 | 0.064 | 0.148 | 0.665 | 0.001 | 0.982 | 0.073 | 0.149 | 0.625 | 0.009 | 0.959 |
| COMT_1_CpG_9 | chr22:19950250 | -0.080 | 0.082 | 0.330 | 0.006 | 0.909 | -0.099 | 0.080 | 0.219 | 0.075 | 0.852 |
| COMT_1_CpG_10 | chr22:19950257 | -0.038 | 0.091 | 0.678 | 0.001 | 0.982 | -0.063 | 0.088 | 0.477 | 0.073 | 0.878 |
| COMT_1_CpG_12 | chr22:19950272 | -0.031 | 0.082 | 0.702 | 0.001 | 0.982 | -0.053 | 0.079 | 0.510 | 0.076 | 0.893 |
| COMT_1_CpG_13 | chr22:19950299 | -0.030 | 0.081 | 0.711 | 0.001 | 0.982 | -0.048 | 0.078 | 0.542 | 0.081 | 0.917 |
| COMT_1_CpG_14and15 | chr22:19950323 | -0.043 | 0.081 | 0.593 | 0.002 | 0.982 | -0.063 | 0.079 | 0.428 | 0.069 | 0.878 |
|  | chr22:19950329 |  |  |  |  |  |  |  |  |  |  |
| COMT_1_CpG_16 | chr22:19950348 | -0.021 | 0.079 | 0.787 | 0.000 | 0.982 | -0.042 | 0.077 | 0.583 | 0.081 | 0.946 |
| COMT_2_CpG_3and4 | chr22:19929115 | 0.040 | 0.038 | 0.288 | 0.007 | 0.909 | 0.039 | 0.038 | 0.308 | 0.017 | 0.878 |
|  | chr22:19929117 |  |  |  |  |  |  |  |  |  |  |
| COMT_2_CpG_5 | chr22:19929131 | 0.016 | 0.013 | 0.214 | 0.010 | 0.909 | 0.015 | 0.013 | 0.248 | 0.015 | 0.852 |
| COMT_2_CpG_6to9 | chr22:19929149 | -0.007 | 0.005 | 0.147 | 0.014 | 0.909 | -0.007 | 0.005 | 0.132 | 0.017 | 0.852 |
|  | chr22:19929152 |  |  |  |  |  |  |  |  |  |  |
|  | chr22:19929154 |  |  |  |  |  |  |  |  |  |  |
|  | chr22:19929156 |  |  |  |  |  |  |  |  |  |  |
| COMT_2_CpG_11to14 | chr22:19929179 | 0.000 | 0.013 | 0.992 | 0.000 | 0.995 | 0.000 | 0.013 | 0.982 | 0.001 | 0.994 |
|  | chr22:19929183 |  |  |  |  |  |  |  |  |  |  |
|  | chr22:19929185 |  |  |  |  |  |  |  |  |  |  |
|  | chr22:19929187 |  |  |  |  |  |  |  |  |  |  |
| COMT_2_CpG_15and16 | chr22:19929198 | -0.005 | 0.011 | 0.663 | 0.001 | 0.982 | -0.004 | 0.011 | 0.721 | 0.046 | 0.966 |
|  | chr22:19929200 |  |  |  |  |  |  |  |  |  |  |
| COMT_2_CpG_17and18 | chr22:19929206 | -0.010 | 0.007 | 0.187 | 0.011 | 0.909 | -0.011 | 0.008 | 0.152 | 0.021 | 0.852 |
|  | chr22:19929211 |  |  |  |  |  |  |  |  |  |  |
| COMT_2_CpG_25and26 | chr22:19929255 | -0.018 | 0.013 | 0.158 | 0.013 | 0.909 | -0.019 | 0.013 | 0.135 | 0.029 | 0.852 |
|  | chr22:19929259 |  |  |  |  |  |  |  |  |  |  |
| COMT_2_CpG_27to29 | chr22:19929264 | 0.002 | 0.014 | 0.880 | 0.000 | 0.982 | 0.003 | 0.014 | 0.822 | 0.012 | 0.966 |
|  | chr22:19929271 |  |  |  |  |  |  |  |  |  |  |
|  | chr22:19929275 |  |  |  |  |  |  |  |  |  |  |
| COMT_2_CpG_31 | chr22:19929287 | 0.006 | 0.007 | 0.422 | 0.004 | 0.909 | 0.005 | 0.007 | 0.454 | 0.006 | 0.878 |
| COMT_2_CpG_32 | chr22:19929302 | 0.005 | 0.006 | 0.460 | 0.004 | 0.909 | 0.005 | 0.006 | 0.466 | 0.006 | 0.878 |
| COMT_2_CpG_33 | chr22:19929307 | -0.084 | 0.044 | 0.061 | 0.023 | 0.909 | -0.077 | 0.044 | 0.087 | 0.045 | 0.852 |
| COMT_2_CpG_34 | chr22:19929313 | 0.010 | 0.055 | 0.855 | 0.000 | 0.982 | 0.011 | 0.055 | 0.844 | 0.004 | 0.966 |
| COMT_2_CpG_35 | chr22:19929322 | 0.012 | 0.015 | 0.439 | 0.004 | 0.909 | 0.012 | 0.016 | 0.428 | 0.005 | 0.878 |
| COMT_2_CpG_36and37 | chr22:19929328 | -0.006 | 0.008 | 0.435 | 0.004 | 0.909 | -0.007 | 0.008 | 0.337 | 0.037 | 0.878 |
|  | chr22:19929331 |  |  |  |  |  |  |  |  |  |  |
| ***FKBP5*** |  |  |  |  |  |  |  |  |  |  |  |
| FKBP5_1_CpG_1 | chr6:35558387 | -0.021 | 0.046 | 0.657 | 0.001 | 0.982 | -0.020 | 0.046 | 0.673 | 0.019 | 0.966 |
| FKBP5_1_CpG_2 | chr6:35558439 | -0.091 | 0.058 | 0.120 | 0.016 | 0.909 | -0.082 | 0.058 | 0.158 | 0.058 | 0.852 |
| FKBP5_1_CpG_3 | chr6:35558489 | -0.062 | 0.038 | 0.109 | 0.017 | 0.909 | -0.066 | 0.039 | 0.092 | 0.025 | 0.852 |
| FKBP5_1_CpG_4 | chr6:35558514 | -0.016 | 0.037 | 0.674 | 0.001 | 0.982 | -0.023 | 0.037 | 0.542 | 0.039 | 0.917 |
| FKBP5_1_CpG_5 | chr6:35558567 | 0.151 | 0.100 | 0.135 | 0.016 | 0.909 | 0.138 | 0.101 | 0.177 | 0.025 | 0.852 |
| ***IL6*** |  |  |  |  |  |  |  |  |  |  |  |
| IL6_1_CpG_1 | chr7:22763499 | 0.066 | 0.057 | 0.247 | 0.009 | 0.909 | 0.078 | 0.056 | 0.165 | 0.054 | 0.852 |
| IL6_1_CpG_3 | chr7:22763600 | 0.036 | 0.049 | 0.462 | 0.004 | 0.909 | 0.048 | 0.049 | 0.331 | 0.037 | 0.878 |
| IL6_1_CpG_4 | chr7:22763717 | -0.059 | 0.045 | 0.191 | 0.011 | 0.909 | -0.061 | 0.046 | 0.181 | 0.013 | 0.852 |
| IL6_1_CpG_5 | chr7:22763745 | -0.074 | 0.062 | 0.233 | 0.009 | 0.909 | -0.071 | 0.063 | 0.261 | 0.013 | 0.874 |
| IL6_1_CpG_6and7 | chr7:22763750 | -0.013 | 0.015 | 0.387 | 0.005 | 0.909 | -0.013 | 0.015 | 0.377 | 0.008 | 0.878 |
|  | chr7:22763752 |  |  |  |  |  |  |  |  |  |  |
| IL6_1_CpG_8 | chr7:22763784 | -0.005 | 0.020 | 0.798 | 0.000 | 0.982 | -0.002 | 0.020 | 0.912 | 0.015 | 0.993 |
| IL6_1_CpG_9 | chr7:22763808 | -0.009 | 0.036 | 0.805 | 0.000 | 0.982 | -0.018 | 0.035 | 0.612 | 0.043 | 0.955 |
| IL6_1_CpG_10and11 | chr7:22763840 | -0.079 | 0.235 | 0.736 | 0.001 | 0.982 | -0.083 | 0.237 | 0.728 | 0.006 | 0.966 |
|  | chr7:22763846 |  |  |  |  |  |  |  |  |  |  |
| IL6_2_CpG_1and2 | chr7:22763911 | -0.004 | 0.005 | 0.482 | 0.003 | 0.909 | -0.004 | 0.005 | 0.434 | 0.026 | 0.878 |
|  | chr7:22763914 |  |  |  |  |  |  |  |  |  |  |
| IL6_2_CpG_3and4 | chr7:22764029 | -0.027 | 0.029 | 0.341 | 0.006 | 0.909 | -0.029 | 0.029 | 0.326 | 0.012 | 0.878 |
|  | chr7:22764031 |  |  |  |  |  |  |  |  |  |  |
| ***IL10*** |  |  |  |  |  |  |  |  |  |  |  |
| IL10_1_CpG_1 | chr1:206940522 | 0.021 | 0.013 | 0.113 | 0.016 | 0.909 | 0.022 | 0.013 | 0.104 | 0.018 | 0.852 |
| IL10_1_CpG_2and3 | chr1:206940451 | -0.013 | 0.021 | 0.527 | 0.003 | 0.941 | -0.011 | 0.021 | 0.591 | 0.008 | 0.949 |
|  | chr1:206940447 |  |  |  |  |  |  |  |  |  |  |
| IL10_1_CpG_4 | chr1:206940364 | -0.021 | 0.017 | 0.223 | 0.010 | 0.909 | -0.020 | 0.017 | 0.245 | 0.014 | 0.852 |
| IL10_1_CpG_5 | chr1:206940327 | -0.068 | 0.043 | 0.113 | 0.016 | 0.909 | -0.066 | 0.043 | 0.126 | 0.018 | 0.852 |
| IL10_1_CpG_6 | chr1:206940311 | 0.195 | 0.117 | 0.098 | 0.018 | 0.909 | 0.204 | 0.118 | 0.086 | 0.027 | 0.852 |
| IL10_2_CpG_1 | chr1:206940215 | -0.014 | 0.016 | 0.375 | 0.005 | 0.909 | -0.017 | 0.016 | 0.267 | 0.035 | 0.878 |
| IL10_2_CpG_2 | chr1:206940208 | -0.007 | 0.032 | 0.837 | 0.000 | 0.982 | -0.007 | 0.032 | 0.835 | 0.000 | 0.966 |
| IL10_2_CpG_3 | chr1:206940167 | -0.020 | 0.015 | 0.189 | 0.011 | 0.909 | -0.019 | 0.015 | 0.199 | 0.014 | 0.852 |
| IL10_2_CpG_4 | chr1:206940003 | 0.021 | 0.042 | 0.611 | 0.002 | 0.982 | 0.033 | 0.041 | 0.426 | 0.071 | 0.878 |
| IL10_2_CpG_5 | chr1:206939984 | 0.026 | 0.021 | 0.224 | 0.010 | 0.909 | 0.031 | 0.021 | 0.135 | 0.066 | 0.852 |
| IL10_2_CpG_6 | chr1:206939954 | 0.029 | 0.028 | 0.302 | 0.007 | 0.909 | 0.036 | 0.028 | 0.191 | 0.069 | 0.852 |
| IL10_2_CpG_7 | chr1:206939896 | 0.031 | 0.041 | 0.448 | 0.004 | 0.909 | 0.041 | 0.040 | 0.302 | 0.074 | 0.878 |
| IL10_2_CpG_9 | chr1:206939813 | 0.026 | 0.037 | 0.478 | 0.003 | 0.909 | 0.036 | 0.036 | 0.320 | 0.063 | 0.878 |
| ***MAOA*** |  |  |  |  |  |  |  |  |  |  |  |
| MAOA_1_CpG_1 | chrX:43514917 | -0.008 | 0.020 | 0.672 | 0.001 | 0.982 | -0.005 | 0.020 | 0.801 | 0.021 | 0.966 |
| MAOA_1_CpG_3 | chrX:43514948 | -0.020 | 0.019 | 0.283 | 0.008 | 0.909 | -0.016 | 0.019 | 0.394 | 0.039 | 0.878 |
| MAOA_1_CpG_4 | chrX:43514973 | -0.011 | 0.021 | 0.591 | 0.002 | 0.982 | -0.007 | 0.021 | 0.745 | 0.030 | 0.966 |
| MAOA_1_CpG_5 | chrX:43514995 | -0.010 | 0.023 | 0.678 | 0.001 | 0.982 | -0.006 | 0.023 | 0.790 | 0.014 | 0.966 |
| MAOA_1_CpG_6 | chrX:43515023 | -0.007 | 0.025 | 0.769 | 0.001 | 0.982 | -0.001 | 0.024 | 0.983 | 0.046 | 0.994 |
| MAOA_1_CpG_7 | chrX:43515066 | -0.017 | 0.025 | 0.492 | 0.003 | 0.909 | -0.011 | 0.025 | 0.645 | 0.036 | 0.963 |
| MAOA_1_CpG_8 | chrX:43515089 | -0.030 | 0.028 | 0.288 | 0.007 | 0.909 | -0.024 | 0.028 | 0.384 | 0.030 | 0.878 |
| MAOA_2_CpG_2and3 | chrX:43515327 | -0.011 | 0.015 | 0.480 | 0.003 | 0.909 | -0.009 | 0.015 | 0.566 | 0.022 | 0.932 |
|  | chrX:43515330 |  |  |  |  |  |  |  |  |  |  |
| MAOA_2_CpG_4and5 | chrX:43515350 | -0.012 | 0.010 | 0.237 | 0.009 | 0.909 | -0.009 | 0.010 | 0.352 | 0.054 | 0.878 |
|  | chrX:43515355 |  |  |  |  |  |  |  |  |  |  |
| MAOA_2_CpG_6 | chrX:43515378 | -0.004 | 0.016 | 0.823 | 0.000 | 0.982 | -0.002 | 0.017 | 0.901 | 0.007 | 0.993 |
| MAOA_2_CpG_7to9 | chrX:43515403 | -0.004 | 0.013 | 0.730 | 0.001 | 0.982 | -0.002 | 0.013 | 0.856 | 0.017 | 0.966 |
|  | chrX:43515413 |  |  |  |  |  |  |  |  |  |  |
|  | chrX:43515419 |  |  |  |  |  |  |  |  |  |  |
| MAOA_2_CpG_10and11 | chrX:43515440 | 0.004 | 0.009 | 0.678 | 0.001 | 0.982 | 0.005 | 0.009 | 0.566 | 0.022 | 0.932 |
|  | chrX:43515445 |  |  |  |  |  |  |  |  |  |  |
| MAOA_2_CpG_12and13 | chrX:43515458 | -0.029 | 0.015 | 0.048 | 0.025 | 0.909 | -0.026 | 0.015 | 0.074 | 0.052 | 0.852 |
|  | chrX:43515468 |  |  |  |  |  |  |  |  |  |  |
| MAOA_2_CpG_18 | chrX:43515545 | -0.002 | 0.014 | 0.889 | 0.000 | 0.982 | 0.001 | 0.014 | 0.938 | 0.029 | 0.994 |
| MAOA_2_CpG_22and23 | chrX:43515617 | 0.001 | 0.016 | 0.959 | 0.000 | 0.982 | 0.002 | 0.017 | 0.915 | 0.003 | 0.993 |
|  | chrX:43515619 |  |  |  |  |  |  |  |  |  |  |
| MAOA_2_CpG_24 | chrX:43515632 | -0.047 | 0.062 | 0.448 | 0.004 | 0.909 | -0.037 | 0.062 | 0.549 | 0.053 | 0.921 |
| MAOA_2_CpG_26 | chrX:43515641 | 0.001 | 0.015 | 0.936 | 0.000 | 0.982 | 0.003 | 0.015 | 0.843 | 0.010 | 0.966 |
| MAOA_2_CpG_27 | chrX:43515647 | -0.049 | 0.052 | 0.348 | 0.006 | 0.909 | -0.046 | 0.053 | 0.386 | 0.010 | 0.878 |
| MAOA_3_CpG_1 | chrX:43515676 | -0.036 | 0.041 | 0.382 | 0.005 | 0.909 | -0.030 | 0.041 | 0.462 | 0.014 | 0.878 |
| MAOA_3_CpG_2 | chrX:43515681 | 0.003 | 0.018 | 0.850 | 0.000 | 0.982 | 0.004 | 0.019 | 0.824 | 0.001 | 0.966 |
| MAOA_3_CpG_3 | chrX:43515763 | -0.030 | 0.159 | 0.848 | 0.000 | 0.982 | -0.041 | 0.160 | 0.796 | 0.007 | 0.966 |
| MAOA_3_CpG_4 | chrX:43515802 | 0.041 | 0.039 | 0.302 | 0.007 | 0.909 | 0.042 | 0.039 | 0.289 | 0.024 | 0.878 |
| MAOA_3_CpG_5 | chrX:43515937 | 0.004 | 0.067 | 0.951 | 0.000 | 0.982 | -0.013 | 0.065 | 0.846 | 0.072 | 0.966 |
| MAOA_3_CpG_6 | chrX:43515991 | -0.013 | 0.058 | 0.822 | 0.000 | 0.982 | -0.027 | 0.057 | 0.629 | 0.056 | 0.959 |
| ***NR3C1*** |  |  |  |  |  |  |  |  |  |  |  |
| NR3C1_1_CpG_3 | chr5:142784324 | 0.004 | 0.009 | 0.607 | 0.002 | 0.982 | 0.004 | 0.009 | 0.615 | 0.002 | 0.955 |
| NR3C1_1_CpG_4 | chr5:142784343 | -0.001 | 0.010 | 0.961 | 0.000 | 0.982 | 0.000 | 0.010 | 0.981 | 0.026 | 0.994 |
| NR3C1_1_CpG_5 | chr5:142784370 | -0.001 | 0.008 | 0.914 | 0.000 | 0.982 | 0.000 | 0.008 | 0.994 | 0.011 | 0.994 |
| NR3C1_1_CpG_6and7 | chr5:142784381 | -0.007 | 0.009 | 0.415 | 0.004 | 0.909 | -0.007 | 0.009 | 0.429 | 0.007 | 0.878 |
|  | chr5:142784383 |  |  |  |  |  |  |  |  |  |  |
| NR3C1_1_CpG_8 | chr5:142784395 | -0.005 | 0.010 | 0.592 | 0.002 | 0.982 | -0.007 | 0.010 | 0.498 | 0.017 | 0.893 |
| NR3C1_1_CpG_9 | chr5:142784413 | 0.030 | 0.013 | 0.021 | 0.034 | 0.807 | 0.029 | 0.013 | 0.022 | 0.065 | 0.705 |
| NR3C1_1_CpG_10 | chr5:142784436 | 0.001 | 0.006 | 0.848 | 0.000 | 0.982 | 0.000 | 0.006 | 0.963 | 0.015 | 0.994 |
| NR3C1_1_CpG_11 | chr5:142784446 | 0.005 | 0.005 | 0.280 | 0.008 | 0.909 | 0.005 | 0.005 | 0.302 | 0.009 | 0.878 |
| NR3C1_1_CpG_12 | chr5:142784463 | 0.029 | 0.038 | 0.445 | 0.004 | 0.909 | 0.027 | 0.038 | 0.478 | 0.006 | 0.878 |
| NR3C1_1_CpG_13 | chr5:142784523 | -0.020 | 0.013 | 0.115 | 0.016 | 0.909 | -0.022 | 0.013 | 0.086 | 0.031 | 0.852 |
| NR3C1_1_CpG_14and15 | chr5:142784586 | -0.001 | 0.009 | 0.873 | 0.000 | 0.982 | -0.002 | 0.009 | 0.859 | 0.015 | 0.966 |
|  | chr5:142784593 |  |  |  |  |  |  |  |  |  |  |
| NR3C1_2_CpG_19and20 | chr5:142783096 | -0.001 | 0.008 | 0.901 | 0.000 | 0.982 | -0.001 | 0.008 | 0.879 | 0.018 | 0.982 |
|  | chr5:142783102 |  |  |  |  |  |  |  |  |  |  |
| NR3C1_2_CpG_22 | chr5:142783113 | -0.001 | 0.007 | 0.860 | 0.000 | 0.982 | -0.002 | 0.007 | 0.810 | 0.019 | 0.966 |
| NR3C1_2_CpG_23and24 | chr5:142783121 | -0.002 | 0.009 | 0.803 | 0.000 | 0.982 | -0.003 | 0.009 | 0.739 | 0.005 | 0.966 |
|  | chr5:142783129 |  |  |  |  |  |  |  |  |  |  |
| NR3C1_2_CpG_27to29 | chr5:142783162 | -0.002 | 0.012 | 0.837 | 0.000 | 0.982 | 0.000 | 0.012 | 0.979 | 0.020 | 0.994 |
|  | chr5:142783165 |  |  |  |  |  |  |  |  |  |  |
|  | chr5:142783168 |  |  |  |  |  |  |  |  |  |  |
| NR3C1_2_CpG_32and33 | chr5:142783190 | 0.010 | 0.147 | 0.946 | 0.000 | 0.982 | 0.026 | 0.147 | 0.859 | 0.029 | 0.966 |
|  | chr5:142783192 |  |  |  |  |  |  |  |  |  |  |
| NR3C1_2_CpG_34and35 | chr5:142783205 | -0.015 | 0.008 | 0.075 | 0.020 | 0.909 | -0.015 | 0.008 | 0.066 | 0.024 | 0.852 |
|  | chr5:142783214 |  |  |  |  |  |  |  |  |  |  |
| NR3C1_2_CpG_37 | chr5:142783222 | 0.026 | 0.022 | 0.243 | 0.009 | 0.909 | 0.027 | 0.022 | 0.233 | 0.015 | 0.852 |
| NR3C1_2_CpG_43to45 | chr5:142783257 | 0.009 | 0.010 | 0.340 | 0.006 | 0.909 | 0.007 | 0.010 | 0.436 | 0.053 | 0.878 |
|  | chr5:142783260 |  |  |  |  |  |  |  |  |  |  |
|  | chr5:142783262 |  |  |  |  |  |  |  |  |  |  |
| NR3C1_2_CpG_46 | chr5:142783272 | 0.022 | 0.029 | 0.441 | 0.004 | 0.909 | 0.022 | 0.029 | 0.444 | 0.016 | 0.878 |
| NR3C1_2_CpG_47and48 | chr5:142783280 | -0.009 | 0.009 | 0.326 | 0.006 | 0.909 | -0.009 | 0.009 | 0.329 | 0.021 | 0.878 |
|  | chr5:142783282 |  |  |  |  |  |  |  |  |  |  |
| NR3C1_2_CpG_49to52 | chr5:142783299 | 0.041 | 0.015 | 0.006 | 0.048 | 0.807 | 0.040 | 0.015 | 0.008 | 0.052 | 0.603 |
|  | chr5:142783303 |  |  |  |  |  |  |  |  |  |  |
|  | chr5:142783310 |  |  |  |  |  |  |  |  |  |  |
|  | chr5:142783314 |  |  |  |  |  |  |  |  |  |  |
| NR3C1_2_CpG_53to58 | chr5:142783322 | 0.014 | 0.016 | 0.398 | 0.005 | 0.909 | 0.014 | 0.017 | 0.412 | 0.008 | 0.878 |
|  | chr5:142783324 |  |  |  |  |  |  |  |  |  |  |
|  | chr5:142783326 |  |  |  |  |  |  |  |  |  |  |
|  | chr5:142783329 |  |  |  |  |  |  |  |  |  |  |
|  | chr5:142783333 |  |  |  |  |  |  |  |  |  |  |
|  | chr5:142783335 |  |  |  |  |  |  |  |  |  |  |
| NR3C1_2_CpG_60 | chr5:142783361 | -0.005 | 0.005 | 0.349 | 0.006 | 0.909 | -0.004 | 0.005 | 0.361 | 0.008 | 0.878 |
| NR3C1_2_CpG_61to63 | chr5:142783380 | 0.057 | 0.031 | 0.066 | 0.022 | 0.909 | 0.055 | 0.031 | 0.078 | 0.024 | 0.852 |
|  | chr5:142783384 |  |  |  |  |  |  |  |  |  |  |
|  | chr5:142783386 |  |  |  |  |  |  |  |  |  |  |
| NR3C1_2_CpG_64to68 | chr5:142783401 | 0.001 | 0.018 | 0.961 | 0.000 | 0.982 | -0.001 | 0.018 | 0.961 | 0.006 | 0.994 |
|  | chr5:142783408 |  |  |  |  |  |  |  |  |  |  |
|  | chr5:142783410 |  |  |  |  |  |  |  |  |  |  |
|  | chr5:142783412 |  |  |  |  |  |  |  |  |  |  |
|  | chr5:142783419 |  |  |  |  |  |  |  |  |  |  |
| NR3C1_2_CpG_69and70 | chr5:142783427 | -0.001 | 0.008 | 0.948 | 0.000 | 0.982 | -0.001 | 0.008 | 0.911 | 0.028 | 0.993 |
|  | chr5:142783433 |  |  |  |  |  |  |  |  |  |  |
| NR3C1_2_CpG_71and72 | chr5:142783436 | -0.004 | 0.009 | 0.682 | 0.001 | 0.982 | -0.003 | 0.009 | 0.736 | 0.012 | 0.966 |
|  | chr5:142783439 |  |  |  |  |  |  |  |  |  |  |
| NR3C1_3_CpG_8 | chr5:142782723 | -0.006 | 0.004 | 0.158 | 0.013 | 0.909 | -0.006 | 0.004 | 0.125 | 0.030 | 0.852 |
| NR3C1_3_CpG_10to13 | chr5:142782703 | -0.013 | 0.010 | 0.203 | 0.010 | 0.909 | -0.012 | 0.011 | 0.244 | 0.016 | 0.852 |
|  | chr5:142782696 |  |  |  |  |  |  |  |  |  |  |
|  | chr5:142782693 |  |  |  |  |  |  |  |  |  |  |
|  | chr5:142782691 |  |  |  |  |  |  |  |  |  |  |
| NR3C1_3_CpG_14 | chr5:142782664 | 0.006 | 0.005 | 0.232 | 0.009 | 0.909 | 0.006 | 0.005 | 0.236 | 0.018 | 0.852 |
| NR3C1_3_CpG_15and16 | chr5:142782633 | -0.001 | 0.005 | 0.822 | 0.000 | 0.982 | -0.001 | 0.005 | 0.859 | 0.005 | 0.966 |
|  | chr5:142782629 |  |  |  |  |  |  |  |  |  |  |
| NR3C1_3_CpG_17 | chr5:142782626 | 0.005 | 0.008 | 0.527 | 0.003 | 0.941 | 0.006 | 0.008 | 0.462 | 0.016 | 0.878 |
| NR3C1_3_CpG_19to21 | chr5:142782609 | -0.006 | 0.007 | 0.398 | 0.005 | 0.909 | -0.006 | 0.007 | 0.434 | 0.011 | 0.878 |
|  | chr5:142782607 |  |  |  |  |  |  |  |  |  |  |
|  | chr5:142782605 |  |  |  |  |  |  |  |  |  |  |
| ***OXTR*** |  |  |  |  |  |  |  |  |  |  |  |
| OXTR_1_CpG_1 | chr3:8809307 | -0.024 | 0.027 | 0.375 | 0.005 | 0.909 | -0.025 | 0.027 | 0.363 | 0.036 | 0.878 |
| OXTR_1_CpG_3and4 | chr3:8809325 | -0.032 | 0.025 | 0.194 | 0.011 | 0.909 | -0.033 | 0.024 | 0.181 | 0.043 | 0.852 |
|  | chr3:8809328 |  |  |  |  |  |  |  |  |  |  |
| OXTR_1_CpG_5and6 | chr3:8809340 | -0.009 | 0.025 | 0.704 | 0.001 | 0.982 | -0.012 | 0.024 | 0.632 | 0.052 | 0.959 |
|  | chr3:8809342 |  |  |  |  |  |  |  |  |  |  |
| OXTR_1_CpG_7to9 | chr3:8809365 | -0.007 | 0.024 | 0.775 | 0.001 | 0.982 | -0.007 | 0.024 | 0.766 | 0.029 | 0.966 |
|  | chr3:8809368 |  |  |  |  |  |  |  |  |  |  |
|  | chr3:8809370 |  |  |  |  |  |  |  |  |  |  |
| OXTR_1_CpG_11and12 | chr3:8809395 | -0.023 | 0.022 | 0.304 | 0.007 | 0.909 | -0.023 | 0.022 | 0.302 | 0.052 | 0.878 |
|  | chr3:8809400 |  |  |  |  |  |  |  |  |  |  |
| OXTR_1_CpG_13to17 | chr3:8809414 | -0.004 | 0.022 | 0.841 | 0.000 | 0.982 | -0.007 | 0.022 | 0.743 | 0.052 | 0.966 |
|  | chr3:8809418 |  |  |  |  |  |  |  |  |  |  |
|  | chr3:8809423 |  |  |  |  |  |  |  |  |  |  |
|  | chr3:8809426 |  |  |  |  |  |  |  |  |  |  |
|  | chr3:8809429 |  |  |  |  |  |  |  |  |  |  |
| OXTR_1_CpG_20 | chr3:8809443 | -0.016 | 0.015 | 0.288 | 0.007 | 0.909 | -0.016 | 0.015 | 0.273 | 0.050 | 0.878 |
| OXTR_1_CpG_21 | chr3:8809465 | -0.036 | 0.026 | 0.170 | 0.012 | 0.909 | -0.037 | 0.026 | 0.167 | 0.036 | 0.852 |
| OXTR_1_CpG_23 | chr3:8809537 | -0.018 | 0.016 | 0.247 | 0.009 | 0.909 | -0.019 | 0.015 | 0.225 | 0.072 | 0.852 |
| OXTR_1_CpG_24and25 | chr3:8809550 | -0.002 | 0.009 | 0.853 | 0.000 | 0.982 | -0.003 | 0.009 | 0.722 | 0.026 | 0.966 |
|  | chr3:8809556 |  |  |  |  |  |  |  |  |  |  |
| OXTR_2_CpG_1 | chr3:8810889 | -0.062 | 0.036 | 0.085 | 0.020 | 0.909 | -0.062 | 0.036 | 0.090 | 0.021 | 0.852 |
| OXTR_2_CpG_2 | chr3:8810874 | -0.014 | 0.021 | 0.490 | 0.003 | 0.909 | -0.017 | 0.021 | 0.415 | 0.014 | 0.878 |
| OXTR_2_CpG_3 | chr3:8810862 | -0.028 | 0.020 | 0.172 | 0.012 | 0.909 | -0.029 | 0.020 | 0.152 | 0.017 | 0.852 |
| OXTR_2_CpG_4 | chr3:8810855 | -0.007 | 0.037 | 0.848 | 0.000 | 0.982 | -0.017 | 0.037 | 0.654 | 0.046 | 0.966 |
| OXTR_2_CpG_5 | chr3:8810832 | 0.016 | 0.035 | 0.641 | 0.001 | 0.982 | 0.012 | 0.035 | 0.726 | 0.016 | 0.966 |
| OXTR_2_CpG_6and7 | chr3:8810807 | 0.043 | 0.037 | 0.247 | 0.009 | 0.909 | 0.043 | 0.037 | 0.250 | 0.012 | 0.852 |
|  | chr3:8810797 |  |  |  |  |  |  |  |  |  |  |
| OXTR_2_CpG_8 | chr3:8810774 | -0.038 | 0.052 | 0.457 | 0.004 | 0.909 | -0.038 | 0.052 | 0.466 | 0.004 | 0.878 |
| OXTR_2_CpG_9 | chr3:8810733 | 0.053 | 0.069 | 0.447 | 0.004 | 0.909 | 0.051 | 0.070 | 0.469 | 0.005 | 0.878 |
| OXTR_2_CpG_10 | chr3:8810708 | -0.008 | 0.020 | 0.685 | 0.001 | 0.982 | -0.006 | 0.020 | 0.749 | 0.039 | 0.966 |
| OXTR_2_CpG_11 | chr3:8810699 | 0.006 | 0.017 | 0.740 | 0.001 | 0.982 | 0.004 | 0.017 | 0.793 | 0.043 | 0.966 |
| OXTR_2_CpG_12and13 | chr3:8810681 | -0.003 | 0.028 | 0.928 | 0.000 | 0.982 | -0.006 | 0.029 | 0.834 | 0.009 | 0.966 |
|  | chr3:8810679 |  |  |  |  |  |  |  |  |  |  |
| OXTR_2_CpG_14 | chr3:8810647 | 0.000 | 0.021 | 0.983 | 0.000 | 0.995 | -0.001 | 0.021 | 0.949 | 0.001 | 0.994 |
| ***SLC6A3*** |  |  |  |  |  |  |  |  |  |  |  |
| SLC6A3_1_CpG_1and2 | chr5:1446585 | 0.007 | 0.025 | 0.777 | 0.001 | 0.982 | 0.006 | 0.026 | 0.822 | 0.003 | 0.966 |
|  | chr5:1446583 |  |  |  |  |  |  |  |  |  |  |
| SLC6A3_1_CpG_3 | chr5:1446545 | -0.014 | 0.024 | 0.549 | 0.002 | 0.961 | -0.016 | 0.024 | 0.518 | 0.004 | 0.899 |
| SLC6A3_1_CpG_4 | chr5:1446537 | 0.079 | 0.031 | 0.012 | 0.040 | 0.807 | 0.080 | 0.032 | 0.012 | 0.041 | 0.603 |
| SLC6A3_1_CpG_5 | chr5:1446517 | 0.009 | 0.022 | 0.674 | 0.001 | 0.982 | 0.008 | 0.022 | 0.726 | 0.007 | 0.966 |
| SLC6A3_1_CpG_7 | chr5:1446498 | -0.017 | 0.013 | 0.169 | 0.012 | 0.909 | -0.019 | 0.013 | 0.124 | 0.030 | 0.852 |
| SLC6A3_1_CpG_8to11 | chr5:1446488 | -0.022 | 0.010 | 0.031 | 0.030 | 0.909 | -0.024 | 0.010 | 0.016 | 0.068 | 0.603 |
|  | chr5:1446485 |  |  |  |  |  |  |  |  |  |  |
|  | chr5:1446478 |  |  |  |  |  |  |  |  |  |  |
|  | chr5:1446474 |  |  |  |  |  |  |  |  |  |  |
| SLC6A3_1_CpG_12 | chr5:1446462 | 0.016 | 0.022 | 0.476 | 0.003 | 0.909 | 0.011 | 0.022 | 0.611 | 0.035 | 0.955 |
| SLC6A3_1_CpG_14and15 | chr5:1446445 | 0.009 | 0.026 | 0.722 | 0.001 | 0.982 | 0.008 | 0.027 | 0.750 | 0.006 | 0.966 |
|  | chr5:1446443 |  |  |  |  |  |  |  |  |  |  |
| SLC6A3_1_CpG_16 | chr5:1446430 | -0.050 | 0.021 | 0.018 | 0.036 | 0.807 | -0.053 | 0.021 | 0.015 | 0.043 | 0.603 |
| SLC6A3_2_CpG_2to4 | chr5:1446371 | 0.026 | 0.011 | 0.019 | 0.035 | 0.807 | 0.027 | 0.011 | 0.016 | 0.043 | 0.603 |
|  | chr5:1446369 |  |  |  |  |  |  |  |  |  |  |
|  | chr5:1446367 |  |  |  |  |  |  |  |  |  |  |
| SLC6A3_2_CpG_5and6 | chr5:1446348 | 0.003 | 0.014 | 0.818 | 0.000 | 0.982 | 0.003 | 0.014 | 0.820 | 0.011 | 0.966 |
|  | chr5:1446344 |  |  |  |  |  |  |  |  |  |  |
| SLC6A3_2_CpG_11 | chr5:1446287 | 0.006 | 0.061 | 0.926 | 0.000 | 0.982 | 0.014 | 0.061 | 0.819 | 0.013 | 0.966 |
| SLC6A3_2_CpG_12and13 | chr5:1446268 | 0.000 | 0.017 | 0.995 | 0.000 | 0.995 | 0.002 | 0.017 | 0.889 | 0.016 | 0.987 |
|  | chr5:1446263 |  |  |  |  |  |  |  |  |  |  |
| SLC6A3_2_CpG_14 | chr5:1446243 | -0.037 | 0.033 | 0.275 | 0.008 | 0.909 | -0.039 | 0.034 | 0.244 | 0.019 | 0.852 |
| SLC6A3_2_CpG_16to18 | chr5:1446232 | -0.008 | 0.013 | 0.546 | 0.002 | 0.961 | -0.009 | 0.013 | 0.506 | 0.006 | 0.893 |
|  | chr5:1446223 |  |  |  |  |  |  |  |  |  |  |
|  | chr5:1446217 |  |  |  |  |  |  |  |  |  |  |
| SLC6A3_2_CpG_21 | chr5:1446188 | -0.003 | 0.021 | 0.900 | 0.000 | 0.982 | -0.009 | 0.021 | 0.680 | 0.056 | 0.966 |
| SLC6A3_2_CpG_22to24 | chr5:1446165 | 0.004 | 0.006 | 0.573 | 0.002 | 0.982 | 0.003 | 0.006 | 0.638 | 0.008 | 0.960 |
|  | chr5:1446161 |  |  |  |  |  |  |  |  |  |  |
|  | chr5:1446156 |  |  |  |  |  |  |  |  |  |  |
| SLC6A3_2_CpG_25and26 | chr5:1446150 | 0.005 | 0.010 | 0.650 | 0.001 | 0.982 | 0.004 | 0.010 | 0.696 | 0.016 | 0.966 |
|  | chr5:1446148 |  |  |  |  |  |  |  |  |  |  |
| SLC6A3_2_CpG_28to30 | chr5:1446121 | 0.006 | 0.011 | 0.630 | 0.002 | 0.982 | 0.004 | 0.012 | 0.718 | 0.019 | 0.966 |
|  | chr5:1446119 |  |  |  |  |  |  |  |  |  |  |
|  | chr5:1446113 |  |  |  |  |  |  |  |  |  |  |
| SLC6A3_2_CpG_32and33 | chr5:1446102 | -0.005 | 0.013 | 0.705 | 0.001 | 0.982 | -0.004 | 0.013 | 0.782 | 0.018 | 0.966 |
|  | chr5:1446099 |  |  |  |  |  |  |  |  |  |  |
| SLC6A3_2_CpG_34 | chr5:1446092 | 0.019 | 0.014 | 0.155 | 0.013 | 0.909 | 0.021 | 0.014 | 0.122 | 0.034 | 0.852 |
| SLC6A3_2_CpG_35to37 | chr5:1446079 | -0.001 | 0.010 | 0.885 | 0.000 | 0.982 | -0.002 | 0.010 | 0.857 | 0.015 | 0.966 |
|  | chr5:1446076 |  |  |  |  |  |  |  |  |  |  |
|  | chr5:1446068 |  |  |  |  |  |  |  |  |  |  |
| SLC6A3_2_CpG_39 | chr5:1446050 | 0.019 | 0.014 | 0.166 | 0.012 | 0.909 | 0.018 | 0.014 | 0.192 | 0.016 | 0.852 |
| SLC6A3_2_CpG_40and41 | chr5:1446043 | -0.015 | 0.016 | 0.328 | 0.006 | 0.909 | -0.019 | 0.016 | 0.227 | 0.046 | 0.852 |
|  | chr5:1446040 |  |  |  |  |  |  |  |  |  |  |
| SLC6A3_2_CpG_42 | chr5:1446026 | -0.007 | 0.009 | 0.399 | 0.005 | 0.909 | -0.008 | 0.009 | 0.383 | 0.014 | 0.878 |
| SLC6A3_2_CpG_43and44 | chr5:1446012 | 0.010 | 0.012 | 0.391 | 0.005 | 0.909 | 0.009 | 0.012 | 0.438 | 0.008 | 0.878 |
|  | chr5:1446010 |  |  |  |  |  |  |  |  |  |  |
| SLC6A3_2_CpG_45 | chr5:1446001 | -0.006 | 0.015 | 0.674 | 0.001 | 0.982 | -0.007 | 0.015 | 0.614 | 0.006 | 0.955 |
| ***SLC6A4*** |  |  |  |  |  |  |  |  |  |  |  |
| SLC6A4_1_CpG_1 | chr17:28563424 | -0.060 | 0.046 | 0.194 | 0.011 | 0.909 | -0.060 | 0.046 | 0.193 | 0.016 | 0.852 |
| SLC6A4_1_CpG_4 | chr17:28563253 | 0.002 | 0.009 | 0.795 | 0.000 | 0.982 | 0.002 | 0.009 | 0.833 | 0.003 | 0.966 |
| SLC6A4_1_CpG_7 | chr17:28563185 | 0.000 | 0.012 | 0.988 | 0.000 | 0.995 | 0.000 | 0.012 | 0.976 | 0.014 | 0.994 |
| SLC6A4_1_CpG_9 | chr17:28563159 | -0.012 | 0.012 | 0.346 | 0.006 | 0.909 | -0.011 | 0.013 | 0.388 | 0.009 | 0.878 |
| SLC6A4_1_CpG_17 | chr17:28563054 | -0.011 | 0.011 | 0.327 | 0.006 | 0.909 | -0.013 | 0.011 | 0.233 | 0.051 | 0.852 |
| SLC6A4_2_CpG_13and14 | chr17:28562914 | 0.001 | 0.007 | 0.916 | 0.000 | 0.982 | 0.000 | 0.007 | 0.990 | 0.005 | 0.994 |
|  | chr17:28562909 |  |  |  |  |  |  |  |  |  |  |
| SLC6A4_2_CpG_15to17 | chr17:28562904 | -0.005 | 0.006 | 0.483 | 0.003 | 0.909 | -0.006 | 0.006 | 0.391 | 0.020 | 0.878 |
|  | chr17:28562902 |  |  |  |  |  |  |  |  |  |  |
|  | chr17:28562888 |  |  |  |  |  |  |  |  |  |  |
| SLC6A4_2_CpG_18 | chr17:28562884 | 0.016 | 0.017 | 0.337 | 0.006 | 0.909 | 0.014 | 0.017 | 0.412 | 0.016 | 0.878 |
| SLC6A4_2_CpG_19 | chr17:28562869 | 0.001 | 0.005 | 0.899 | 0.000 | 0.982 | 0.000 | 0.005 | 0.944 | 0.009 | 0.994 |
| SLC6A4_2_CpG_20and21 | chr17:28562863 | -0.005 | 0.004 | 0.247 | 0.009 | 0.909 | -0.004 | 0.004 | 0.296 | 0.015 | 0.878 |
|  | chr17:28562861 |  |  |  |  |  |  |  |  |  |  |
| SLC6A4_2_CpG_22to25 | chr17:28562855 | -0.009 | 0.007 | 0.196 | 0.011 | 0.909 | -0.010 | 0.007 | 0.190 | 0.032 | 0.852 |
|  | chr17:28562853 |  |  |  |  |  |  |  |  |  |  |
|  | chr17:28562849 |  |  |  |  |  |  |  |  |  |  |
|  | chr17:28562847 |  |  |  |  |  |  |  |  |  |  |
| SLC6A4_2_CpG_26 | chr17:28562826 | 0.000 | 0.005 | 0.940 | 0.000 | 0.982 | -0.001 | 0.005 | 0.827 | 0.078 | 0.966 |
| SLC6A4_2_CpG_28and29 | chr17:28562786 | -0.010 | 0.005 | 0.034 | 0.029 | 0.909 | -0.010 | 0.005 | 0.038 | 0.066 | 0.852 |
|  | chr17:28562783 |  |  |  |  |  |  |  |  |  |  |
| SLC6A4_3_CpG_1and2 | chr17:28562751 | -0.013 | 0.008 | 0.126 | 0.015 | 0.909 | -0.013 | 0.008 | 0.110 | 0.020 | 0.852 |
|  | chr17:28562749 |  |  |  |  |  |  |  |  |  |  |
| SLC6A4_3_CpG_3to8 | chr17:28562737 | 0.029 | 0.025 | 0.260 | 0.008 | 0.909 | 0.026 | 0.025 | 0.304 | 0.024 | 0.878 |
|  | chr17:28562733 |  |  |  |  |  |  |  |  |  |  |
|  | chr17:28562731 |  |  |  |  |  |  |  |  |  |  |
|  | chr17:28562728 |  |  |  |  |  |  |  |  |  |  |
|  | chr17:28562725 |  |  |  |  |  |  |  |  |  |  |
|  | chr17:28562717 |  |  |  |  |  |  |  |  |  |  |
| SLC6A4_3_CpG_9to12 | chr17:28562706 | -0.013 | 0.014 | 0.369 | 0.005 | 0.909 | -0.017 | 0.014 | 0.216 | 0.088 | 0.852 |
|  | chr17:28562703 |  |  |  |  |  |  |  |  |  |  |
|  | chr17:28562700 |  |  |  |  |  |  |  |  |  |  |
|  | chr17:28562691 |  |  |  |  |  |  |  |  |  |  |
| SLC6A4_3_CpG_13and14 | chr17:28562685 | 0.003 | 0.011 | 0.782 | 0.001 | 0.982 | 0.000 | 0.010 | 0.977 | 0.102 | 0.994 |
|  | chr17:28562683 |  |  |  |  |  |  |  |  |  |  |
| SLC6A4_3_CpG_15 | chr17:28562672 | -0.004 | 0.012 | 0.755 | 0.001 | 0.982 | -0.008 | 0.011 | 0.485 | 0.138 | 0.883 |
| SLC6A4_3_CpG_16 | chr17:28562659 | -0.005 | 0.004 | 0.267 | 0.008 | 0.909 | -0.005 | 0.004 | 0.218 | 0.029 | 0.852 |
| SLC6A4_3_CpG_22 | chr17:28562596 | -0.033 | 0.023 | 0.151 | 0.013 | 0.909 | -0.035 | 0.023 | 0.125 | 0.022 | 0.852 |
| SLC6A4_3_CpG_23and24 | chr17:28562572 | 0.001 | 0.014 | 0.952 | 0.000 | 0.982 | 0.003 | 0.014 | 0.853 | 0.010 | 0.966 |
|  | chr17:28562567 |  |  |  |  |  |  |  |  |  |  |
| SLC6A4_3_CpG_27and28 | chr17:28562536 | -0.002 | 0.016 | 0.909 | 0.000 | 0.982 | -0.009 | 0.015 | 0.538 | 0.156 | 0.917 |
|  | chr17:28562529 |  |  |  |  |  |  |  |  |  |  |
| SLC6A4_3_CpG_29 | chr17:28562521 | -0.004 | 0.050 | 0.940 | 0.000 | 0.982 | -0.009 | 0.051 | 0.855 | 0.012 | 0.966 |
| SLC6A4_3_CpG_30 | chr17:28562507 | -0.042 | 0.050 | 0.407 | 0.004 | 0.909 | -0.048 | 0.051 | 0.346 | 0.016 | 0.878 |
| SLC6A4_3_CpG_31to33 | chr17:28562499 | -0.018 | 0.014 | 0.200 | 0.011 | 0.909 | -0.020 | 0.014 | 0.138 | 0.052 | 0.852 |
|  | chr17:28562492 |  |  |  |  |  |  |  |  |  |  |
|  | chr17:28562489 |  |  |  |  |  |  |  |  |  |  |
| SLC6A4_3_CpG_34 | chr17:28562474 | 0.012 | 0.018 | 0.495 | 0.003 | 0.909 | 0.010 | 0.018 | 0.585 | 0.025 | 0.946 |
| SLC6A4_3_CpG_35 | chr17:28562465 | -0.043 | 0.038 | 0.264 | 0.008 | 0.909 | -0.045 | 0.039 | 0.247 | 0.011 | 0.852 |
| SLC6A4_3_CpG_36 | chr17:28562435 | -0.029 | 0.015 | 0.046 | 0.026 | 0.909 | -0.032 | 0.015 | 0.027 | 0.060 | 0.724 |
| SLC6A4_3_CpG_38 | chr17:28562412 | -0.022 | 0.013 | 0.098 | 0.018 | 0.909 | -0.024 | 0.013 | 0.070 | 0.041 | 0.852 |
| SLC6A4_3_CpG_39 | chr17:28562401 | 0.006 | 0.025 | 0.798 | 0.000 | 0.982 | 0.002 | 0.025 | 0.941 | 0.021 | 0.994 |
| SLC6A4_3_CpG_40and41 | chr17:28562392 | -0.042 | 0.028 | 0.137 | 0.014 | 0.909 | -0.048 | 0.028 | 0.080 | 0.056 | 0.852 |
|  | chr17:28562388 |  |  |  |  |  |  |  |  |  |  |
| *Note.* Based on GRCh37/hg19 coordinates. | |  |  |  |  |  |  |  |  |  |  |
