# Supplementary material for: Association between child maltreatment and depressive symptoms in emerging adulthood: The mediating and moderating roles of DNA methylation
Source: PLoS One. 2023 Jan 12;18(1):e0280203. doi: 10.1371/journal.pone.0280203 (PMC9836296; doi:10.1371/journal.pone.0280203)
Supplement: S3 Table — Based on GRCh37/hg19 coordinates. (DOCX) [file pone.0280203.s003.docx]

| **S3 Table. Associations between depressive symptoms and DNA methylation.** | | | | | | | | | | | |
| --- | --- | --- | --- | --- | --- | --- | --- | --- | --- | --- | --- |
| CpG Name | Position | DNA Methylation | | | | | | | | | |
|  |  | Unadjusted Models | | | | | Adjusted Models | | | | |
|  |  | *B* | *SE* | *p* | *R2* | *FDR* | *B* | *SE* | *p* | *R2* | *FDR* |
| ***COMT*** |  |  |  |  |  |  |  |  |  |  |  |
| COMT_1_CpG_3 | chr22:19950055 | -0.086 | 0.080 | 0.284 | 0.007 | 0.759 | -0.057 | 0.082 | 0.485 | 0.040 | 0.759 |
| COMT_1_CpG_4 | chr22:19950064 | -0.116 | 0.096 | 0.232 | 0.009 | 0.750 | -0.059 | 0.096 | 0.540 | 0.079 | 0.750 |
| COMT_1_CpG_5 | chr22:19950158 | -0.168 | 0.100 | 0.095 | 0.018 | 0.680 | -0.108 | 0.100 | 0.282 | 0.079 | 0.680 |
| COMT_1_CpG_7 | chr22:19950222 | -0.224 | 0.156 | 0.154 | 0.013 | 0.688 | -0.190 | 0.159 | 0.235 | 0.046 | 0.688 |
| COMT_1_CpG_8 | chr22:19950236 | 0.059 | 0.190 | 0.755 | 0.001 | 0.864 | 0.098 | 0.196 | 0.616 | 0.009 | 0.864 |
| COMT_1_CpG_9 | chr22:19950250 | -0.141 | 0.105 | 0.179 | 0.012 | 0.698 | -0.078 | 0.105 | 0.461 | 0.070 | 0.698 |
| COMT_1_CpG_10 | chr22:19950257 | -0.125 | 0.116 | 0.284 | 0.007 | 0.759 | -0.062 | 0.116 | 0.592 | 0.072 | 0.759 |
| COMT_1_CpG_12 | chr22:19950272 | -0.156 | 0.104 | 0.136 | 0.014 | 0.688 | -0.094 | 0.104 | 0.368 | 0.079 | 0.688 |
| COMT_1_CpG_13 | chr22:19950299 | -0.142 | 0.103 | 0.171 | 0.012 | 0.695 | -0.070 | 0.103 | 0.497 | 0.081 | 0.695 |
| COMT_1_CpG_14and15 | chr22:19950323 | -0.152 | 0.103 | 0.143 | 0.014 | 0.688 | -0.093 | 0.103 | 0.369 | 0.071 | 0.688 |
|  | chr22:19950329 |  |  |  |  |  |  |  |  |  |  |
| COMT_1_CpG_16 | chr22:19950348 | -0.147 | 0.101 | 0.146 | 0.014 | 0.688 | -0.084 | 0.101 | 0.403 | 0.083 | 0.688 |
| COMT_2_CpG_3and4 | chr22:19929115 | -0.027 | 0.049 | 0.581 | 0.002 | 0.864 | -0.013 | 0.050 | 0.791 | 0.011 | 0.864 |
|  | chr22:19929117 |  |  |  |  |  |  |  |  |  |  |
| COMT_2_CpG_5 | chr22:19929131 | 0.004 | 0.017 | 0.830 | 0.000 | 0.916 | 0.002 | 0.017 | 0.889 | 0.007 | 0.916 |
| COMT_2_CpG_6to9 | chr22:19929149 | -0.002 | 0.006 | 0.720 | 0.001 | 0.864 | -0.002 | 0.006 | 0.737 | 0.003 | 0.864 |
|  | chr22:19929152 |  |  |  |  |  |  |  |  |  |  |
|  | chr22:19929154 |  |  |  |  |  |  |  |  |  |  |
|  | chr22:19929156 |  |  |  |  |  |  |  |  |  |  |
| COMT_2_CpG_11to14 | chr22:19929179 | -0.005 | 0.016 | 0.743 | 0.001 | 0.864 | -0.006 | 0.017 | 0.733 | 0.002 | 0.864 |
|  | chr22:19929183 |  |  |  |  |  |  |  |  |  |  |
|  | chr22:19929185 |  |  |  |  |  |  |  |  |  |  |
|  | chr22:19929187 |  |  |  |  |  |  |  |  |  |  |
| COMT_2_CpG_15and16 | chr22:19929198 | -0.004 | 0.014 | 0.752 | 0.001 | 0.864 | -0.014 | 0.014 | 0.333 | 0.051 | 0.864 |
|  | chr22:19929200 |  |  |  |  |  |  |  |  |  |  |
| COMT_2_CpG_17and18 | chr22:19929206 | -0.006 | 0.010 | 0.567 | 0.002 | 0.864 | -0.006 | 0.010 | 0.578 | 0.009 | 0.864 |
|  | chr22:19929211 |  |  |  |  |  |  |  |  |  |  |
| COMT_2_CpG_25and26 | chr22:19929255 | -0.016 | 0.017 | 0.345 | 0.006 | 0.822 | -0.021 | 0.017 | 0.214 | 0.024 | 0.822 |
|  | chr22:19929259 |  |  |  |  |  |  |  |  |  |  |
| COMT_2_CpG_27to29 | chr22:19929264 | 0.022 | 0.018 | 0.217 | 0.010 | 0.750 | 0.018 | 0.018 | 0.331 | 0.018 | 0.750 |
|  | chr22:19929271 |  |  |  |  |  |  |  |  |  |  |
|  | chr22:19929275 |  |  |  |  |  |  |  |  |  |  |
| COMT_2_CpG_31 | chr22:19929287 | 0.008 | 0.009 | 0.399 | 0.005 | 0.864 | 0.008 | 0.009 | 0.372 | 0.007 | 0.864 |
| COMT_2_CpG_32 | chr22:19929302 | -0.005 | 0.008 | 0.566 | 0.002 | 0.864 | -0.004 | 0.008 | 0.655 | 0.004 | 0.864 |
| COMT_2_CpG_33 | chr22:19929307 | -0.024 | 0.058 | 0.677 | 0.001 | 0.864 | -0.045 | 0.059 | 0.450 | 0.030 | 0.864 |
| COMT_2_CpG_34 | chr22:19929313 | 0.024 | 0.070 | 0.731 | 0.001 | 0.864 | 0.013 | 0.073 | 0.861 | 0.004 | 0.864 |
| COMT_2_CpG_35 | chr22:19929322 | -0.003 | 0.020 | 0.896 | 0.000 | 0.935 | -0.004 | 0.020 | 0.857 | 0.001 | 0.935 |
| COMT_2_CpG_36and37 | chr22:19929328 | 0.004 | 0.010 | 0.681 | 0.001 | 0.864 | 0.002 | 0.010 | 0.873 | 0.031 | 0.864 |
|  | chr22:19929331 |  |  |  |  |  |  |  |  |  |  |
| ***FKBP5*** |  |  |  |  |  |  |  |  |  |  |  |
| FKBP5_1_CpG_1 | chr6:35558387 | 0.037 | 0.060 | 0.536 | 0.002 | 0.864 | 0.015 | 0.061 | 0.806 | 0.018 | 0.864 |
| FKBP5_1_CpG_2 | chr6:35558439 | -0.020 | 0.075 | 0.791 | 0.000 | 0.889 | -0.064 | 0.076 | 0.400 | 0.049 | 0.889 |
| FKBP5_1_CpG_3 | chr6:35558489 | -0.044 | 0.050 | 0.374 | 0.005 | 0.840 | -0.036 | 0.051 | 0.482 | 0.010 | 0.840 |
| FKBP5_1_CpG_4 | chr6:35558514 | -0.039 | 0.048 | 0.414 | 0.004 | 0.864 | -0.019 | 0.048 | 0.700 | 0.037 | 0.864 |
| FKBP5_1_CpG_5 | chr6:35558567 | 0.249 | 0.128 | 0.054 | 0.026 | 0.602 | 0.237 | 0.132 | 0.075 | 0.035 | 0.602 |
| ***IL6*** |  |  |  |  |  |  |  |  |  |  |  |
| IL6_1_CpG_1 | chr7:22763499 | 0.102 | 0.073 | 0.162 | 0.013 | 0.688 | 0.072 | 0.074 | 0.330 | 0.047 | 0.688 |
| IL6_1_CpG_3 | chr7:22763600 | 0.068 | 0.063 | 0.286 | 0.007 | 0.759 | 0.055 | 0.064 | 0.395 | 0.036 | 0.759 |
| IL6_1_CpG_4 | chr7:22763717 | 0.018 | 0.058 | 0.753 | 0.001 | 0.864 | 0.018 | 0.060 | 0.768 | 0.001 | 0.864 |
| IL6_1_CpG_5 | chr7:22763745 | -0.057 | 0.080 | 0.475 | 0.003 | 0.864 | -0.055 | 0.083 | 0.510 | 0.007 | 0.864 |
| IL6_1_CpG_6and7 | chr7:22763750 | -0.009 | 0.019 | 0.653 | 0.001 | 0.864 | -0.006 | 0.020 | 0.766 | 0.004 | 0.864 |
|  | chr7:22763752 |  |  |  |  |  |  |  |  |  |  |
| IL6_1_CpG_8 | chr7:22763784 | 0.003 | 0.026 | 0.905 | 0.000 | 0.940 | -0.002 | 0.027 | 0.929 | 0.015 | 0.940 |
| IL6_1_CpG_9 | chr7:22763808 | 0.024 | 0.046 | 0.603 | 0.002 | 0.864 | 0.040 | 0.046 | 0.386 | 0.046 | 0.864 |
| IL6_1_CpG_10and11 | chr7:22763840 | -0.229 | 0.301 | 0.448 | 0.004 | 0.864 | -0.300 | 0.311 | 0.336 | 0.011 | 0.864 |
|  | chr7:22763846 |  |  |  |  |  |  |  |  |  |  |
| IL6_2_CpG_1and2 | chr7:22763911 | 0.007 | 0.007 | 0.330 | 0.006 | 0.822 | 0.004 | 0.007 | 0.526 | 0.025 | 0.822 |
|  | chr7:22763914 |  |  |  |  |  |  |  |  |  |  |
| IL6_2_CpG_3and4 | chr7:22764029 | -0.082 | 0.037 | 0.028 | 0.033 | 0.597 | -0.093 | 0.038 | 0.015 | 0.046 | 0.597 |
|  | chr7:22764031 |  |  |  |  |  |  |  |  |  |  |
| ***IL10*** |  |  |  |  |  |  |  |  |  |  |  |
| IL10_1_CpG_1 | chr1:206940522 | 0.019 | 0.017 | 0.269 | 0.008 | 0.759 | 0.019 | 0.018 | 0.272 | 0.009 | 0.759 |
| IL10_1_CpG_2and3 | chr1:206940451 | -0.032 | 0.027 | 0.237 | 0.009 | 0.753 | -0.036 | 0.028 | 0.198 | 0.017 | 0.753 |
|  | chr1:206940447 |  |  |  |  |  |  |  |  |  |  |
| IL10_1_CpG_4 | chr1:206940364 | -0.004 | 0.022 | 0.847 | 0.000 | 0.918 | -0.002 | 0.022 | 0.946 | 0.005 | 0.918 |
| IL10_1_CpG_5 | chr1:206940327 | -0.045 | 0.055 | 0.414 | 0.004 | 0.864 | -0.044 | 0.057 | 0.446 | 0.007 | 0.864 |
| IL10_1_CpG_6 | chr1:206940311 | 0.211 | 0.151 | 0.163 | 0.013 | 0.688 | 0.185 | 0.156 | 0.237 | 0.017 | 0.688 |
| IL10_2_CpG_1 | chr1:206940215 | -0.030 | 0.020 | 0.137 | 0.014 | 0.688 | -0.028 | 0.021 | 0.173 | 0.039 | 0.688 |
| IL10_2_CpG_2 | chr1:206940208 | -0.026 | 0.041 | 0.530 | 0.003 | 0.864 | -0.027 | 0.042 | 0.528 | 0.003 | 0.864 |
| IL10_2_CpG_3 | chr1:206940167 | 0.011 | 0.019 | 0.574 | 0.002 | 0.864 | 0.008 | 0.020 | 0.680 | 0.004 | 0.864 |
| IL10_2_CpG_4 | chr1:206940003 | 0.110 | 0.053 | 0.040 | 0.027 | 0.597 | 0.084 | 0.054 | 0.119 | 0.082 | 0.597 |
| IL10_2_CpG_5 | chr1:206939984 | 0.033 | 0.027 | 0.227 | 0.009 | 0.750 | 0.021 | 0.027 | 0.444 | 0.055 | 0.750 |
| IL10_2_CpG_6 | chr1:206939954 | 0.068 | 0.036 | 0.060 | 0.023 | 0.636 | 0.051 | 0.036 | 0.160 | 0.070 | 0.636 |
| IL10_2_CpG_7 | chr1:206939896 | 0.085 | 0.052 | 0.103 | 0.017 | 0.680 | 0.056 | 0.052 | 0.289 | 0.075 | 0.680 |
| IL10_2_CpG_9 | chr1:206939813 | 0.072 | 0.047 | 0.126 | 0.015 | 0.688 | 0.052 | 0.047 | 0.269 | 0.065 | 0.688 |
| ***MAOA*** |  |  |  |  |  |  |  |  |  |  |  |
| MAOA_1_CpG_1 | chrX:43514917 | -0.003 | 0.026 | 0.910 | 0.000 | 0.940 | -0.008 | 0.026 | 0.750 | 0.021 | 0.940 |
| MAOA_1_CpG_3 | chrX:43514948 | -0.014 | 0.024 | 0.573 | 0.002 | 0.864 | -0.018 | 0.025 | 0.464 | 0.038 | 0.864 |
| MAOA_1_CpG_4 | chrX:43514973 | -0.014 | 0.027 | 0.619 | 0.002 | 0.864 | -0.015 | 0.028 | 0.596 | 0.031 | 0.864 |
| MAOA_1_CpG_5 | chrX:43514995 | -0.005 | 0.030 | 0.881 | 0.000 | 0.929 | -0.006 | 0.031 | 0.858 | 0.014 | 0.929 |
| MAOA_1_CpG_6 | chrX:43515023 | -0.006 | 0.032 | 0.856 | 0.000 | 0.918 | -0.011 | 0.033 | 0.731 | 0.047 | 0.918 |
| MAOA_1_CpG_7 | chrX:43515066 | 0.003 | 0.032 | 0.918 | 0.000 | 0.943 | -0.002 | 0.033 | 0.955 | 0.034 | 0.943 |
| MAOA_1_CpG_8 | chrX:43515089 | -0.022 | 0.036 | 0.540 | 0.002 | 0.864 | -0.025 | 0.037 | 0.502 | 0.028 | 0.864 |
| MAOA_2_CpG_2and3 | chrX:43515327 | -0.017 | 0.019 | 0.386 | 0.005 | 0.848 | -0.024 | 0.020 | 0.224 | 0.029 | 0.848 |
|  | chrX:43515330 |  |  |  |  |  |  |  |  |  |  |
| MAOA_2_CpG_4and5 | chrX:43515350 | 0.012 | 0.013 | 0.346 | 0.006 | 0.822 | 0.009 | 0.013 | 0.472 | 0.052 | 0.822 |
|  | chrX:43515355 |  |  |  |  |  |  |  |  |  |  |
| MAOA_2_CpG_6 | chrX:43515378 | -0.010 | 0.021 | 0.642 | 0.001 | 0.864 | -0.014 | 0.022 | 0.536 | 0.010 | 0.864 |
| MAOA_2_CpG_7to9 | chrX:43515403 | -0.015 | 0.016 | 0.364 | 0.005 | 0.840 | -0.018 | 0.017 | 0.296 | 0.024 | 0.840 |
|  | chrX:43515413 |  |  |  |  |  |  |  |  |  |  |
|  | chrX:43515419 |  |  |  |  |  |  |  |  |  |  |
| MAOA_2_CpG_10and11 | chrX:43515440 | -0.004 | 0.012 | 0.759 | 0.001 | 0.864 | -0.002 | 0.012 | 0.869 | 0.020 | 0.864 |
|  | chrX:43515445 |  |  |  |  |  |  |  |  |  |  |
| MAOA_2_CpG_12and13 | chrX:43515458 | -0.013 | 0.019 | 0.492 | 0.003 | 0.864 | -0.020 | 0.019 | 0.315 | 0.038 | 0.864 |
|  | chrX:43515468 |  |  |  |  |  |  |  |  |  |  |
| MAOA_2_CpG_18 | chrX:43515545 | -0.006 | 0.018 | 0.760 | 0.001 | 0.864 | -0.007 | 0.018 | 0.717 | 0.030 | 0.864 |
| MAOA_2_CpG_22and23 | chrX:43515617 | -0.025 | 0.021 | 0.231 | 0.009 | 0.750 | -0.025 | 0.022 | 0.254 | 0.012 | 0.750 |
|  | chrX:43515619 |  |  |  |  |  |  |  |  |  |  |
| MAOA_2_CpG_24 | chrX:43515632 | -0.012 | 0.081 | 0.883 | 0.000 | 0.929 | -0.063 | 0.082 | 0.441 | 0.055 | 0.929 |
| MAOA_2_CpG_26 | chrX:43515641 | 0.010 | 0.020 | 0.618 | 0.002 | 0.864 | 0.011 | 0.020 | 0.572 | 0.012 | 0.864 |
| MAOA_2_CpG_27 | chrX:43515647 | -0.110 | 0.068 | 0.107 | 0.018 | 0.680 | -0.108 | 0.070 | 0.128 | 0.022 | 0.680 |
| MAOA_3_CpG_1 | chrX:43515676 | 0.099 | 0.053 | 0.065 | 0.024 | 0.649 | 0.102 | 0.055 | 0.063 | 0.035 | 0.649 |
| MAOA_3_CpG_2 | chrX:43515681 | 0.005 | 0.024 | 0.848 | 0.000 | 0.918 | 0.005 | 0.024 | 0.843 | 0.001 | 0.918 |
| MAOA_3_CpG_3 | chrX:43515763 | -0.104 | 0.202 | 0.607 | 0.002 | 0.864 | -0.077 | 0.210 | 0.715 | 0.007 | 0.864 |
| MAOA_3_CpG_4 | chrX:43515802 | 0.003 | 0.051 | 0.949 | 0.000 | 0.969 | 0.022 | 0.052 | 0.670 | 0.018 | 0.969 |
| MAOA_3_CpG_5 | chrX:43515937 | -0.104 | 0.086 | 0.228 | 0.009 | 0.750 | -0.052 | 0.086 | 0.544 | 0.074 | 0.750 |
| MAOA_3_CpG_6 | chrX:43515991 | -0.071 | 0.074 | 0.340 | 0.006 | 0.822 | -0.036 | 0.074 | 0.627 | 0.056 | 0.822 |
| ***NR3C1*** |  |  |  |  |  |  |  |  |  |  |  |
| NR3C1_1_CpG_3 | chr5:142784324 | -0.008 | 0.011 | 0.486 | 0.003 | 0.864 | -0.008 | 0.011 | 0.485 | 0.003 | 0.864 |
| NR3C1_1_CpG_4 | chr5:142784343 | 0.026 | 0.013 | 0.051 | 0.025 | 0.602 | 0.021 | 0.013 | 0.123 | 0.041 | 0.602 |
| NR3C1_1_CpG_5 | chr5:142784370 | 0.004 | 0.010 | 0.689 | 0.001 | 0.864 | 0.006 | 0.011 | 0.572 | 0.013 | 0.864 |
| NR3C1_1_CpG_6and7 | chr5:142784381 | 0.001 | 0.011 | 0.956 | 0.000 | 0.971 | 0.002 | 0.011 | 0.860 | 0.003 | 0.971 |
|  | chr5:142784383 |  |  |  |  |  |  |  |  |  |  |
| NR3C1_1_CpG_8 | chr5:142784395 | -0.007 | 0.013 | 0.585 | 0.002 | 0.864 | -0.008 | 0.013 | 0.519 | 0.017 | 0.864 |
| NR3C1_1_CpG_9 | chr5:142784413 | -0.006 | 0.017 | 0.706 | 0.001 | 0.864 | 0.002 | 0.017 | 0.884 | 0.033 | 0.864 |
| NR3C1_1_CpG_10 | chr5:142784436 | 0.000 | 0.008 | 0.980 | 0.000 | 0.988 | -0.001 | 0.008 | 0.923 | 0.016 | 0.988 |
| NR3C1_1_CpG_11 | chr5:142784446 | 0.003 | 0.006 | 0.636 | 0.001 | 0.864 | 0.003 | 0.007 | 0.648 | 0.003 | 0.864 |
| NR3C1_1_CpG_12 | chr5:142784463 | -0.022 | 0.048 | 0.657 | 0.001 | 0.864 | -0.017 | 0.050 | 0.727 | 0.003 | 0.864 |
| NR3C1_1_CpG_13 | chr5:142784523 | -0.024 | 0.017 | 0.142 | 0.014 | 0.688 | -0.027 | 0.017 | 0.120 | 0.027 | 0.688 |
| NR3C1_1_CpG_14and15 | chr5:142784586 | -0.020 | 0.011 | 0.084 | 0.019 | 0.680 | -0.016 | 0.012 | 0.158 | 0.028 | 0.680 |
|  | chr5:142784593 |  |  |  |  |  |  |  |  |  |  |
| NR3C1_2_CpG_19and20 | chr5:142783096 | -0.012 | 0.011 | 0.258 | 0.008 | 0.759 | -0.009 | 0.011 | 0.440 | 0.022 | 0.759 |
|  | chr5:142783102 |  |  |  |  |  |  |  |  |  |  |
| NR3C1_2_CpG_22 | chr5:142783113 | 0.003 | 0.009 | 0.741 | 0.001 | 0.864 | 0.000 | 0.010 | 0.999 | 0.019 | 0.864 |
| NR3C1_2_CpG_23and24 | chr5:142783121 | -0.006 | 0.012 | 0.608 | 0.002 | 0.864 | -0.006 | 0.012 | 0.646 | 0.006 | 0.864 |
|  | chr5:142783129 |  |  |  |  |  |  |  |  |  |  |
| NR3C1_2_CpG_27to29 | chr5:142783162 | -0.004 | 0.015 | 0.783 | 0.000 | 0.885 | -0.005 | 0.015 | 0.755 | 0.021 | 0.885 |
|  | chr5:142783165 |  |  |  |  |  |  |  |  |  |  |
|  | chr5:142783168 |  |  |  |  |  |  |  |  |  |  |
| NR3C1_2_CpG_32and33 | chr5:142783190 | -0.028 | 0.194 | 0.885 | 0.000 | 0.929 | -0.094 | 0.195 | 0.632 | 0.031 | 0.929 |
|  | chr5:142783192 |  |  |  |  |  |  |  |  |  |  |
| NR3C1_2_CpG_34and35 | chr5:142783205 | -0.008 | 0.011 | 0.481 | 0.003 | 0.864 | -0.008 | 0.011 | 0.498 | 0.005 | 0.864 |
|  | chr5:142783214 |  |  |  |  |  |  |  |  |  |  |
| NR3C1_2_CpG_37 | chr5:142783222 | -0.001 | 0.029 | 0.983 | 0.000 | 0.988 | -0.007 | 0.029 | 0.803 | 0.006 | 0.988 |
| NR3C1_2_CpG_43to45 | chr5:142783257 | 0.012 | 0.012 | 0.348 | 0.006 | 0.822 | 0.019 | 0.012 | 0.119 | 0.065 | 0.822 |
|  | chr5:142783260 |  |  |  |  |  |  |  |  |  |  |
|  | chr5:142783262 |  |  |  |  |  |  |  |  |  |  |
| NR3C1_2_CpG_46 | chr5:142783272 | -0.020 | 0.037 | 0.579 | 0.002 | 0.864 | -0.034 | 0.038 | 0.374 | 0.018 | 0.864 |
| NR3C1_2_CpG_47and48 | chr5:142783280 | 0.009 | 0.011 | 0.421 | 0.004 | 0.864 | 0.005 | 0.012 | 0.639 | 0.016 | 0.864 |
|  | chr5:142783282 |  |  |  |  |  |  |  |  |  |  |
| NR3C1_2_CpG_49to52 | chr5:142783299 | 0.043 | 0.019 | 0.027 | 0.031 | 0.597 | 0.048 | 0.020 | 0.016 | 0.044 | 0.597 |
|  | chr5:142783303 |  |  |  |  |  |  |  |  |  |  |
|  | chr5:142783310 |  |  |  |  |  |  |  |  |  |  |
|  | chr5:142783314 |  |  |  |  |  |  |  |  |  |  |
| NR3C1_2_CpG_53to58 | chr5:142783322 | 0.000 | 0.021 | 0.993 | 0.000 | 0.993 | 0.004 | 0.022 | 0.852 | 0.004 | 0.993 |
|  | chr5:142783324 |  |  |  |  |  |  |  |  |  |  |
|  | chr5:142783326 |  |  |  |  |  |  |  |  |  |  |
|  | chr5:142783329 |  |  |  |  |  |  |  |  |  |  |
|  | chr5:142783333 |  |  |  |  |  |  |  |  |  |  |
|  | chr5:142783335 |  |  |  |  |  |  |  |  |  |  |
| NR3C1_2_CpG_60 | chr5:142783361 | -0.009 | 0.006 | 0.130 | 0.015 | 0.688 | -0.009 | 0.006 | 0.157 | 0.016 | 0.688 |
| NR3C1_2_CpG_61to63 | chr5:142783380 | 0.099 | 0.039 | 0.013 | 0.039 | 0.597 | 0.107 | 0.041 | 0.009 | 0.048 | 0.597 |
|  | chr5:142783384 |  |  |  |  |  |  |  |  |  |  |
|  | chr5:142783386 |  |  |  |  |  |  |  |  |  |  |
| NR3C1_2_CpG_64to68 | chr5:142783401 | 0.009 | 0.023 | 0.683 | 0.001 | 0.864 | 0.010 | 0.024 | 0.659 | 0.007 | 0.864 |
|  | chr5:142783408 |  |  |  |  |  |  |  |  |  |  |
|  | chr5:142783410 |  |  |  |  |  |  |  |  |  |  |
|  | chr5:142783412 |  |  |  |  |  |  |  |  |  |  |
|  | chr5:142783419 |  |  |  |  |  |  |  |  |  |  |
| NR3C1_2_CpG_69and70 | chr5:142783427 | 0.004 | 0.011 | 0.711 | 0.001 | 0.864 | -0.001 | 0.011 | 0.943 | 0.028 | 0.864 |
|  | chr5:142783433 |  |  |  |  |  |  |  |  |  |  |
| NR3C1_2_CpG_71and72 | chr5:142783436 | -0.011 | 0.011 | 0.316 | 0.007 | 0.806 | -0.015 | 0.012 | 0.184 | 0.023 | 0.806 |
|  | chr5:142783439 |  |  |  |  |  |  |  |  |  |  |
| NR3C1_3_CpG_8 | chr5:142782723 | 0.002 | 0.005 | 0.640 | 0.001 | 0.864 | 0.002 | 0.005 | 0.766 | 0.015 | 0.864 |
| NR3C1_3_CpG_10to13 | chr5:142782703 | -0.005 | 0.013 | 0.733 | 0.001 | 0.864 | -0.006 | 0.014 | 0.664 | 0.009 | 0.864 |
|  | chr5:142782696 |  |  |  |  |  |  |  |  |  |  |
|  | chr5:142782693 |  |  |  |  |  |  |  |  |  |  |
|  | chr5:142782691 |  |  |  |  |  |  |  |  |  |  |
| NR3C1_3_CpG_14 | chr5:142782664 | -0.010 | 0.006 | 0.102 | 0.017 | 0.680 | -0.009 | 0.006 | 0.164 | 0.022 | 0.680 |
| NR3C1_3_CpG_15and16 | chr5:142782633 | -0.005 | 0.007 | 0.435 | 0.004 | 0.864 | -0.004 | 0.007 | 0.513 | 0.008 | 0.864 |
|  | chr5:142782629 |  |  |  |  |  |  |  |  |  |  |
| NR3C1_3_CpG_17 | chr5:142782626 | 0.005 | 0.010 | 0.663 | 0.001 | 0.864 | 0.002 | 0.011 | 0.861 | 0.012 | 0.864 |
| NR3C1_3_CpG_19to21 | chr5:142782609 | -0.008 | 0.010 | 0.420 | 0.004 | 0.864 | -0.010 | 0.010 | 0.291 | 0.015 | 0.864 |
|  | chr5:142782607 |  |  |  |  |  |  |  |  |  |  |
|  | chr5:142782605 |  |  |  |  |  |  |  |  |  |  |
| ***OXTR*** |  |  |  |  |  |  |  |  |  |  |  |
| OXTR_1_CpG_1 | chr3:8809307 | -0.041 | 0.035 | 0.246 | 0.009 | 0.759 | -0.024 | 0.036 | 0.493 | 0.034 | 0.759 |
| OXTR_1_CpG_3and4 | chr3:8809325 | -0.036 | 0.032 | 0.262 | 0.008 | 0.759 | -0.020 | 0.032 | 0.526 | 0.034 | 0.759 |
|  | chr3:8809328 |  |  |  |  |  |  |  |  |  |  |
| OXTR_1_CpG_5and6 | chr3:8809340 | -0.044 | 0.031 | 0.164 | 0.013 | 0.688 | -0.025 | 0.032 | 0.433 | 0.054 | 0.688 |
|  | chr3:8809342 |  |  |  |  |  |  |  |  |  |  |
| OXTR_1_CpG_7to9 | chr3:8809365 | -0.030 | 0.031 | 0.337 | 0.006 | 0.822 | -0.016 | 0.032 | 0.616 | 0.030 | 0.822 |
|  | chr3:8809368 |  |  |  |  |  |  |  |  |  |  |
|  | chr3:8809370 |  |  |  |  |  |  |  |  |  |  |
| OXTR_1_CpG_11and12 | chr3:8809395 | -0.026 | 0.029 | 0.366 | 0.005 | 0.840 | -0.009 | 0.029 | 0.744 | 0.046 | 0.840 |
|  | chr3:8809400 |  |  |  |  |  |  |  |  |  |  |
| OXTR_1_CpG_13to17 | chr3:8809414 | -0.011 | 0.028 | 0.689 | 0.001 | 0.864 | 0.007 | 0.029 | 0.807 | 0.052 | 0.864 |
|  | chr3:8809418 |  |  |  |  |  |  |  |  |  |  |
|  | chr3:8809423 |  |  |  |  |  |  |  |  |  |  |
|  | chr3:8809426 |  |  |  |  |  |  |  |  |  |  |
|  | chr3:8809429 |  |  |  |  |  |  |  |  |  |  |
| OXTR_1_CpG_20 | chr3:8809443 | -0.019 | 0.019 | 0.310 | 0.007 | 0.800 | -0.009 | 0.019 | 0.653 | 0.044 | 0.800 |
| OXTR_1_CpG_21 | chr3:8809465 | -0.011 | 0.034 | 0.743 | 0.001 | 0.864 | 0.004 | 0.035 | 0.918 | 0.024 | 0.864 |
| OXTR_1_CpG_23 | chr3:8809537 | -0.014 | 0.020 | 0.499 | 0.003 | 0.864 | 0.001 | 0.020 | 0.968 | 0.063 | 0.864 |
| OXTR_1_CpG_24and25 | chr3:8809550 | -0.005 | 0.011 | 0.683 | 0.001 | 0.864 | -0.001 | 0.011 | 0.931 | 0.025 | 0.864 |
|  | chr3:8809556 |  |  |  |  |  |  |  |  |  |  |
| OXTR_2_CpG_1 | chr3:8810889 | -0.066 | 0.047 | 0.158 | 0.014 | 0.688 | -0.074 | 0.048 | 0.125 | 0.018 | 0.688 |
| OXTR_2_CpG_2 | chr3:8810874 | -0.037 | 0.027 | 0.166 | 0.012 | 0.688 | -0.035 | 0.027 | 0.203 | 0.021 | 0.688 |
| OXTR_2_CpG_3 | chr3:8810862 | -0.046 | 0.026 | 0.075 | 0.020 | 0.680 | -0.050 | 0.027 | 0.063 | 0.026 | 0.680 |
| OXTR_2_CpG_4 | chr3:8810855 | 0.069 | 0.048 | 0.147 | 0.014 | 0.688 | 0.072 | 0.048 | 0.135 | 0.059 | 0.688 |
| OXTR_2_CpG_5 | chr3:8810832 | 0.015 | 0.045 | 0.731 | 0.001 | 0.864 | 0.030 | 0.046 | 0.516 | 0.018 | 0.864 |
| OXTR_2_CpG_6and7 | chr3:8810807 | 0.054 | 0.047 | 0.257 | 0.008 | 0.759 | 0.066 | 0.049 | 0.179 | 0.016 | 0.759 |
|  | chr3:8810797 |  |  |  |  |  |  |  |  |  |  |
| OXTR_2_CpG_8 | chr3:8810774 | 0.026 | 0.066 | 0.701 | 0.001 | 0.864 | 0.029 | 0.069 | 0.674 | 0.001 | 0.864 |
| OXTR_2_CpG_9 | chr3:8810733 | 0.049 | 0.088 | 0.577 | 0.002 | 0.864 | 0.050 | 0.091 | 0.582 | 0.003 | 0.864 |
| OXTR_2_CpG_10 | chr3:8810708 | -0.034 | 0.025 | 0.178 | 0.012 | 0.698 | -0.024 | 0.026 | 0.364 | 0.044 | 0.698 |
| OXTR_2_CpG_11 | chr3:8810699 | -0.046 | 0.021 | 0.032 | 0.029 | 0.597 | -0.035 | 0.022 | 0.107 | 0.058 | 0.597 |
| OXTR_2_CpG_12and13 | chr3:8810681 | -0.059 | 0.036 | 0.102 | 0.017 | 0.680 | -0.059 | 0.037 | 0.118 | 0.025 | 0.680 |
|  | chr3:8810679 |  |  |  |  |  |  |  |  |  |  |
| OXTR_2_CpG_14 | chr3:8810647 | -0.035 | 0.027 | 0.192 | 0.011 | 0.718 | -0.035 | 0.027 | 0.205 | 0.012 | 0.718 |
| ***SLC6A3*** |  |  |  |  |  |  |  |  |  |  |  |
| SLC6A3_1_CpG_1and2 | chr5:1446585 | -0.039 | 0.032 | 0.229 | 0.009 | 0.750 | -0.039 | 0.034 | 0.248 | 0.011 | 0.750 |
|  | chr5:1446583 |  |  |  |  |  |  |  |  |  |  |
| SLC6A3_1_CpG_3 | chr5:1446545 | 0.060 | 0.031 | 0.051 | 0.024 | 0.602 | 0.063 | 0.032 | 0.046 | 0.027 | 0.602 |
| SLC6A3_1_CpG_4 | chr5:1446537 | -0.015 | 0.041 | 0.713 | 0.001 | 0.864 | -0.012 | 0.042 | 0.773 | 0.001 | 0.864 |
| SLC6A3_1_CpG_5 | chr5:1446517 | -0.018 | 0.028 | 0.535 | 0.003 | 0.864 | -0.013 | 0.029 | 0.650 | 0.007 | 0.864 |
| SLC6A3_1_CpG_7 | chr5:1446498 | -0.033 | 0.016 | 0.041 | 0.027 | 0.597 | -0.035 | 0.016 | 0.033 | 0.044 | 0.597 |
| SLC6A3_1_CpG_8to11 | chr5:1446488 | -0.009 | 0.013 | 0.501 | 0.003 | 0.864 | -0.010 | 0.013 | 0.464 | 0.035 | 0.864 |
|  | chr5:1446485 |  |  |  |  |  |  |  |  |  |  |
|  | chr5:1446478 |  |  |  |  |  |  |  |  |  |  |
|  | chr5:1446474 |  |  |  |  |  |  |  |  |  |  |
| SLC6A3_1_CpG_12 | chr5:1446462 | -0.061 | 0.029 | 0.035 | 0.028 | 0.597 | -0.054 | 0.029 | 0.064 | 0.055 | 0.597 |
| SLC6A3_1_CpG_14and15 | chr5:1446445 | -0.013 | 0.034 | 0.713 | 0.001 | 0.864 | -0.020 | 0.035 | 0.576 | 0.008 | 0.864 |
|  | chr5:1446443 |  |  |  |  |  |  |  |  |  |  |
| SLC6A3_1_CpG_16 | chr5:1446430 | -0.065 | 0.027 | 0.018 | 0.036 | 0.597 | -0.064 | 0.028 | 0.023 | 0.038 | 0.597 |
| SLC6A3_2_CpG_2to4 | chr5:1446371 | 0.006 | 0.015 | 0.681 | 0.001 | 0.864 | 0.008 | 0.015 | 0.580 | 0.008 | 0.864 |
|  | chr5:1446369 |  |  |  |  |  |  |  |  |  |  |
|  | chr5:1446367 |  |  |  |  |  |  |  |  |  |  |
| SLC6A3_2_CpG_5and6 | chr5:1446348 | -0.026 | 0.018 | 0.137 | 0.014 | 0.688 | -0.022 | 0.018 | 0.219 | 0.020 | 0.688 |
|  | chr5:1446344 |  |  |  |  |  |  |  |  |  |  |
| SLC6A3_2_CpG_11 | chr5:1446287 | 0.128 | 0.077 | 0.099 | 0.018 | 0.680 | 0.123 | 0.080 | 0.125 | 0.027 | 0.680 |
| SLC6A3_2_CpG_12and13 | chr5:1446268 | 0.007 | 0.022 | 0.759 | 0.001 | 0.864 | 0.001 | 0.022 | 0.956 | 0.015 | 0.864 |
|  | chr5:1446263 |  |  |  |  |  |  |  |  |  |  |
| SLC6A3_2_CpG_14 | chr5:1446243 | -0.027 | 0.042 | 0.524 | 0.003 | 0.864 | -0.038 | 0.044 | 0.380 | 0.015 | 0.864 |
| SLC6A3_2_CpG_16to18 | chr5:1446232 | -0.011 | 0.017 | 0.511 | 0.003 | 0.864 | -0.010 | 0.017 | 0.577 | 0.005 | 0.864 |
|  | chr5:1446223 |  |  |  |  |  |  |  |  |  |  |
|  | chr5:1446217 |  |  |  |  |  |  |  |  |  |  |
| SLC6A3_2_CpG_21 | chr5:1446188 | 0.007 | 0.027 | 0.810 | 0.000 | 0.899 | 0.018 | 0.027 | 0.512 | 0.058 | 0.899 |
| SLC6A3_2_CpG_22to24 | chr5:1446165 | -0.004 | 0.008 | 0.598 | 0.002 | 0.864 | -0.003 | 0.008 | 0.682 | 0.008 | 0.864 |
|  | chr5:1446161 |  |  |  |  |  |  |  |  |  |  |
|  | chr5:1446156 |  |  |  |  |  |  |  |  |  |  |
| SLC6A3_2_CpG_25and26 | chr5:1446150 | 0.007 | 0.013 | 0.576 | 0.002 | 0.864 | 0.013 | 0.013 | 0.353 | 0.021 | 0.864 |
|  | chr5:1446148 |  |  |  |  |  |  |  |  |  |  |
| SLC6A3_2_CpG_28to30 | chr5:1446121 | -0.003 | 0.015 | 0.865 | 0.000 | 0.923 | 0.002 | 0.015 | 0.882 | 0.018 | 0.923 |
|  | chr5:1446119 |  |  |  |  |  |  |  |  |  |  |
|  | chr5:1446113 |  |  |  |  |  |  |  |  |  |  |
| SLC6A3_2_CpG_32and33 | chr5:1446102 | 0.018 | 0.016 | 0.264 | 0.008 | 0.759 | 0.024 | 0.017 | 0.157 | 0.031 | 0.759 |
|  | chr5:1446099 |  |  |  |  |  |  |  |  |  |  |
| SLC6A3_2_CpG_34 | chr5:1446092 | 0.022 | 0.018 | 0.213 | 0.010 | 0.750 | 0.028 | 0.018 | 0.126 | 0.033 | 0.750 |
| SLC6A3_2_CpG_35to37 | chr5:1446079 | -0.007 | 0.013 | 0.569 | 0.002 | 0.864 | -0.003 | 0.013 | 0.824 | 0.015 | 0.864 |
|  | chr5:1446076 |  |  |  |  |  |  |  |  |  |  |
|  | chr5:1446068 |  |  |  |  |  |  |  |  |  |  |
| SLC6A3_2_CpG_39 | chr5:1446050 | 0.025 | 0.018 | 0.151 | 0.013 | 0.688 | 0.029 | 0.018 | 0.117 | 0.021 | 0.688 |
| SLC6A3_2_CpG_40and41 | chr5:1446043 | -0.023 | 0.020 | 0.262 | 0.008 | 0.759 | -0.016 | 0.020 | 0.440 | 0.040 | 0.759 |
|  | chr5:1446040 |  |  |  |  |  |  |  |  |  |  |
| SLC6A3_2_CpG_42 | chr5:1446026 | -0.018 | 0.011 | 0.104 | 0.017 | 0.680 | -0.016 | 0.011 | 0.168 | 0.022 | 0.680 |
| SLC6A3_2_CpG_43and44 | chr5:1446012 | 0.004 | 0.015 | 0.800 | 0.000 | 0.893 | 0.005 | 0.016 | 0.773 | 0.005 | 0.893 |
|  | chr5:1446010 |  |  |  |  |  |  |  |  |  |  |
| SLC6A3_2_CpG_45 | chr5:1446001 | 0.033 | 0.019 | 0.074 | 0.021 | 0.680 | 0.037 | 0.019 | 0.055 | 0.028 | 0.680 |
| ***SLC6A4*** |  |  |  |  |  |  |  |  |  |  |  |
| SLC6A4_1_CpG_1 | chr17:28563424 | 0.021 | 0.059 | 0.720 | 0.001 | 0.864 | 0.010 | 0.061 | 0.875 | 0.005 | 0.864 |
| SLC6A4_1_CpG_4 | chr17:28563253 | -0.012 | 0.011 | 0.276 | 0.008 | 0.759 | -0.012 | 0.012 | 0.322 | 0.009 | 0.759 |
| SLC6A4_1_CpG_7 | chr17:28563185 | 0.006 | 0.016 | 0.703 | 0.001 | 0.864 | 0.012 | 0.016 | 0.478 | 0.017 | 0.864 |
| SLC6A4_1_CpG_9 | chr17:28563159 | -0.012 | 0.016 | 0.454 | 0.004 | 0.864 | -0.013 | 0.016 | 0.414 | 0.008 | 0.864 |
| SLC6A4_1_CpG_17 | chr17:28563054 | -0.007 | 0.014 | 0.614 | 0.002 | 0.864 | -0.013 | 0.014 | 0.355 | 0.047 | 0.864 |
| SLC6A4_2_CpG_13and14 | chr17:28562914 | -0.005 | 0.009 | 0.545 | 0.002 | 0.864 | -0.006 | 0.009 | 0.531 | 0.008 | 0.864 |
|  | chr17:28562909 |  |  |  |  |  |  |  |  |  |  |
| SLC6A4_2_CpG_15to17 | chr17:28562904 | -0.005 | 0.008 | 0.508 | 0.003 | 0.864 | -0.004 | 0.008 | 0.617 | 0.017 | 0.864 |
|  | chr17:28562902 |  |  |  |  |  |  |  |  |  |  |
|  | chr17:28562888 |  |  |  |  |  |  |  |  |  |  |
| SLC6A4_2_CpG_18 | chr17:28562884 | -0.012 | 0.022 | 0.573 | 0.002 | 0.864 | -0.012 | 0.022 | 0.594 | 0.014 | 0.864 |
| SLC6A4_2_CpG_19 | chr17:28562869 | 0.007 | 0.007 | 0.304 | 0.007 | 0.795 | 0.006 | 0.007 | 0.407 | 0.013 | 0.795 |
| SLC6A4_2_CpG_20and21 | chr17:28562863 | 0.003 | 0.005 | 0.512 | 0.003 | 0.864 | 0.004 | 0.005 | 0.498 | 0.011 | 0.864 |
|  | chr17:28562861 |  |  |  |  |  |  |  |  |  |  |
| SLC6A4_2_CpG_22to25 | chr17:28562855 | -0.007 | 0.009 | 0.452 | 0.004 | 0.864 | -0.003 | 0.010 | 0.726 | 0.022 | 0.864 |
|  | chr17:28562853 |  |  |  |  |  |  |  |  |  |  |
|  | chr17:28562849 |  |  |  |  |  |  |  |  |  |  |
|  | chr17:28562847 |  |  |  |  |  |  |  |  |  |  |
| SLC6A4_2_CpG_26 | chr17:28562826 | -0.014 | 0.006 | 0.033 | 0.029 | 0.597 | -0.009 | 0.006 | 0.154 | 0.090 | 0.597 |
| SLC6A4_2_CpG_28and29 | chr17:28562786 | -0.013 | 0.006 | 0.024 | 0.033 | 0.597 | -0.011 | 0.006 | 0.069 | 0.060 | 0.597 |
|  | chr17:28562783 |  |  |  |  |  |  |  |  |  |  |
| SLC6A4_3_CpG_1and2 | chr17:28562751 | -0.021 | 0.010 | 0.048 | 0.025 | 0.602 | -0.022 | 0.011 | 0.039 | 0.031 | 0.602 |
|  | chr17:28562749 |  |  |  |  |  |  |  |  |  |  |
| SLC6A4_3_CpG_3to8 | chr17:28562737 | -0.006 | 0.033 | 0.852 | 0.000 | 0.918 | -0.016 | 0.034 | 0.629 | 0.019 | 0.918 |
|  | chr17:28562733 |  |  |  |  |  |  |  |  |  |  |
|  | chr17:28562731 |  |  |  |  |  |  |  |  |  |  |
|  | chr17:28562728 |  |  |  |  |  |  |  |  |  |  |
|  | chr17:28562725 |  |  |  |  |  |  |  |  |  |  |
|  | chr17:28562717 |  |  |  |  |  |  |  |  |  |  |
| SLC6A4_3_CpG_9to12 | chr17:28562706 | -0.050 | 0.018 | 0.005 | 0.050 | 0.597 | -0.042 | 0.018 | 0.020 | 0.111 | 0.597 |
|  | chr17:28562703 |  |  |  |  |  |  |  |  |  |  |
|  | chr17:28562700 |  |  |  |  |  |  |  |  |  |  |
|  | chr17:28562691 |  |  |  |  |  |  |  |  |  |  |
| SLC6A4_3_CpG_13and14 | chr17:28562685 | -0.005 | 0.014 | 0.689 | 0.001 | 0.864 | 0.005 | 0.013 | 0.726 | 0.103 | 0.864 |
|  | chr17:28562683 |  |  |  |  |  |  |  |  |  |  |
| SLC6A4_3_CpG_15 | chr17:28562672 | -0.019 | 0.015 | 0.214 | 0.010 | 0.750 | -0.006 | 0.015 | 0.685 | 0.136 | 0.750 |
| SLC6A4_3_CpG_16 | chr17:28562659 | -0.003 | 0.005 | 0.531 | 0.003 | 0.864 | -0.002 | 0.006 | 0.778 | 0.020 | 0.864 |
| SLC6A4_3_CpG_22 | chr17:28562596 | -0.066 | 0.029 | 0.022 | 0.034 | 0.597 | -0.065 | 0.030 | 0.030 | 0.038 | 0.597 |
| SLC6A4_3_CpG_23and24 | chr17:28562572 | 0.004 | 0.018 | 0.836 | 0.000 | 0.918 | 0.005 | 0.019 | 0.810 | 0.010 | 0.918 |
|  | chr17:28562567 |  |  |  |  |  |  |  |  |  |  |
| SLC6A4_3_CpG_27and28 | chr17:28562536 | -0.035 | 0.020 | 0.083 | 0.020 | 0.680 | -0.023 | 0.019 | 0.230 | 0.162 | 0.680 |
|  | chr17:28562529 |  |  |  |  |  |  |  |  |  |  |
| SLC6A4_3_CpG_29 | chr17:28562521 | -0.070 | 0.064 | 0.278 | 0.008 | 0.759 | -0.057 | 0.067 | 0.396 | 0.017 | 0.759 |
| SLC6A4_3_CpG_30 | chr17:28562507 | -0.058 | 0.065 | 0.373 | 0.005 | 0.840 | -0.069 | 0.066 | 0.302 | 0.018 | 0.840 |
| SLC6A4_3_CpG_31to33 | chr17:28562499 | -0.041 | 0.018 | 0.021 | 0.034 | 0.597 | -0.034 | 0.018 | 0.058 | 0.061 | 0.597 |
|  | chr17:28562492 |  |  |  |  |  |  |  |  |  |  |
|  | chr17:28562489 |  |  |  |  |  |  |  |  |  |  |
| SLC6A4_3_CpG_34 | chr17:28562474 | 0.012 | 0.023 | 0.607 | 0.002 | 0.864 | 0.022 | 0.024 | 0.366 | 0.029 | 0.864 |
| SLC6A4_3_CpG_35 | chr17:28562465 | -0.038 | 0.050 | 0.442 | 0.004 | 0.864 | -0.036 | 0.051 | 0.487 | 0.005 | 0.864 |
| SLC6A4_3_CpG_36 | chr17:28562435 | -0.025 | 0.019 | 0.190 | 0.011 | 0.718 | -0.020 | 0.019 | 0.304 | 0.036 | 0.718 |
| SLC6A4_3_CpG_38 | chr17:28562412 | -0.015 | 0.017 | 0.379 | 0.005 | 0.842 | -0.010 | 0.017 | 0.561 | 0.022 | 0.842 |
| SLC6A4_3_CpG_39 | chr17:28562401 | 0.023 | 0.032 | 0.477 | 0.003 | 0.864 | 0.026 | 0.033 | 0.436 | 0.025 | 0.864 |
| SLC6A4_3_CpG_40and41 | chr17:28562392 | -0.014 | 0.036 | 0.705 | 0.001 | 0.864 | -0.003 | 0.037 | 0.927 | 0.037 | 0.864 |
|  | chr17:28562388 |  |  |  |  |  |  |  |  |  |  |
| *Note.* Based on GRCh37/hg19 coordinates. | |  |  |  |  |  |  |  |  |  |  |
