# Supplementary figures and images for: Association between child maltreatment and depressive symptoms in emerging adulthood: The mediating and moderating roles of DNA methylation
Source: PLoS One. 2023 Jan 12;18(1):e0280203. doi: 10.1371/journal.pone.0280203 (PMC9836296; doi:10.1371/journal.pone.0280203)

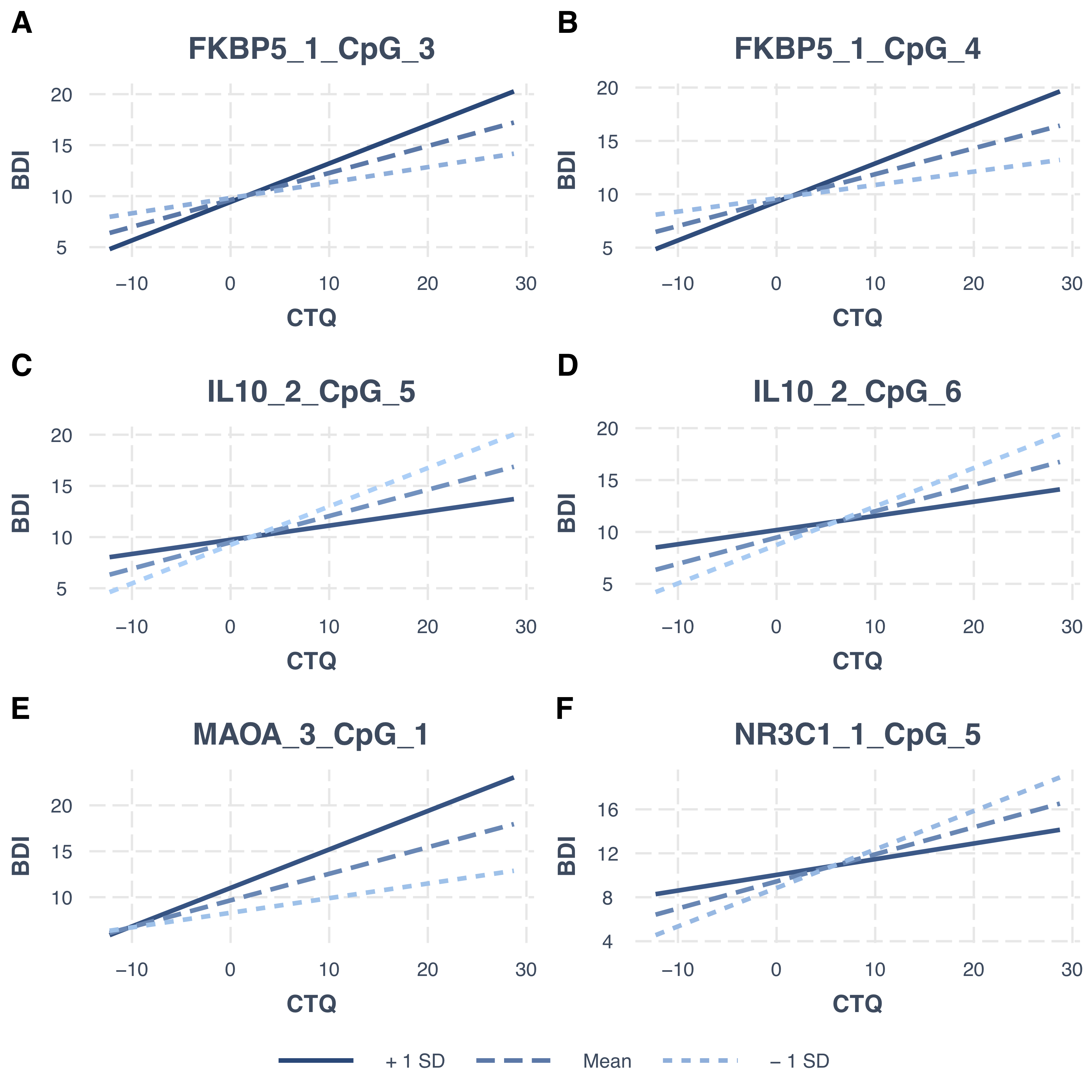

Supplement: S1 Fig — (A) Adjusted model for FKBP5_1_CpG_3. (B) Adjusted model for FKBP5_1_CpG_4. (C) Adjusted model for IL10_2_CpG_5. (D) Adjusted model for IL10_2_CpG_6. (E) Adjusted model for MAOA_3_CpG_1. (F) Adjusted model for NR3C1_1_CpG_5. (TIF) [file pone.0280203.s004.tif]

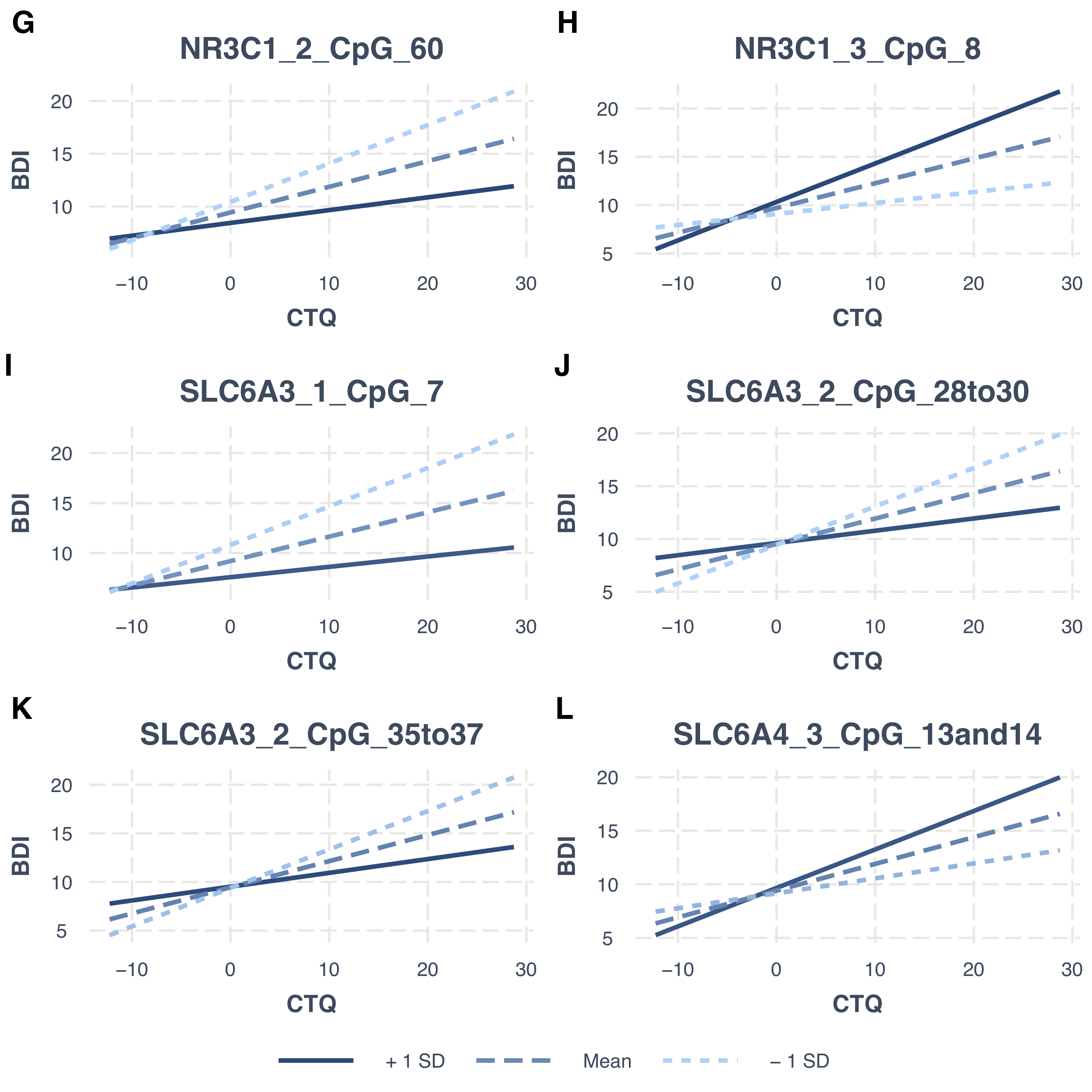

Supplement: S2 Fig — (G) Adjusted model for NR3C1_2_CpG_60. (H) Adjusted model for NR3C1_3_CpG_8. (I) Adjusted model for SLC6A3_1_CpG_7. (J) Adjusted model for SLC6A3_2_CpG_28to30. (K) Adjusted model for SLC6A3_2_CpG_35to37. (L) Adjusted model for SLC6A4_3_CpG_13and14. (TIF) [file pone.0280203.s005.tif]

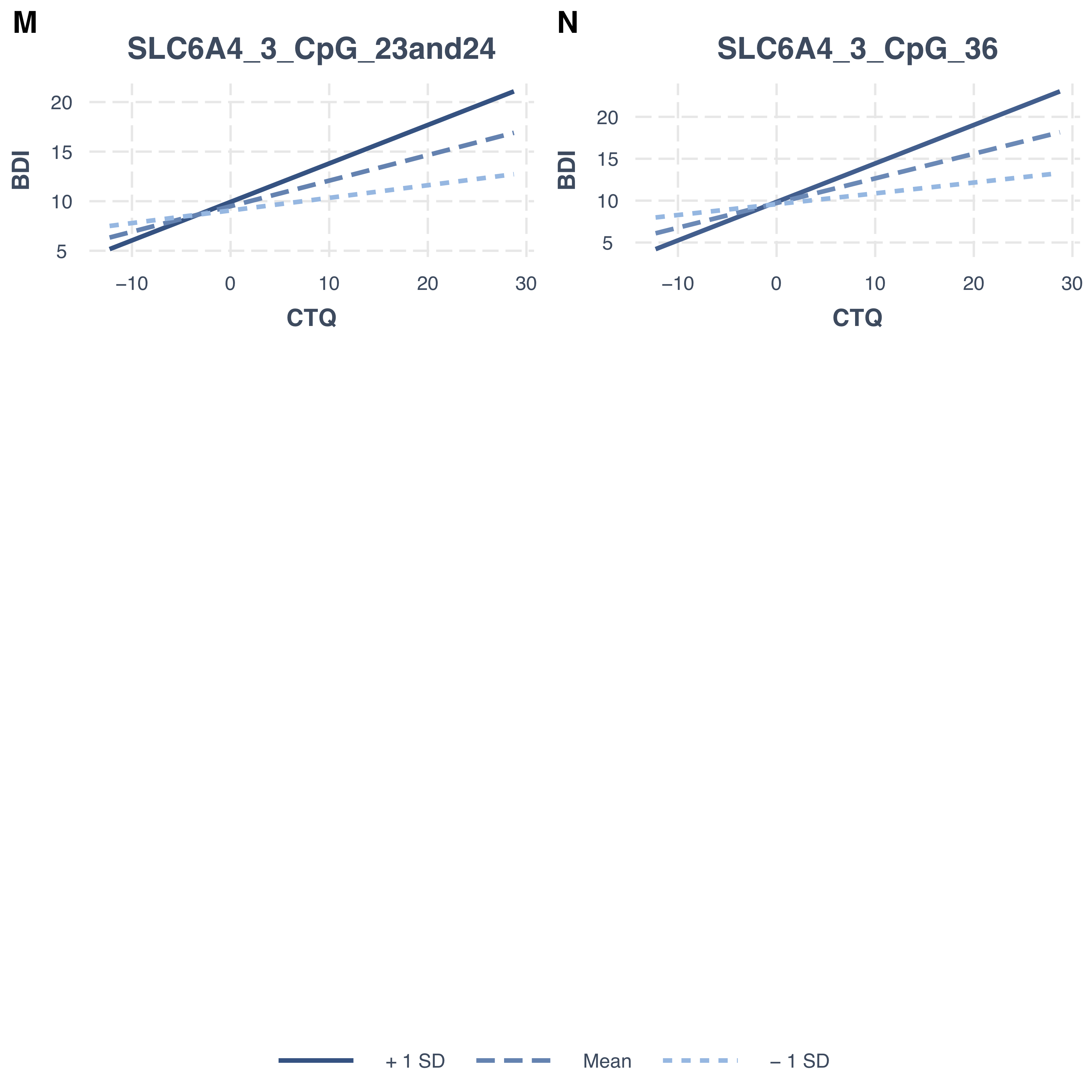

Supplement: S3 Fig — (M) Adjusted model for SLC6A4_3_CpG_23and24. (N) Adjusted model for SLC6A4_3_CpG_36. (TIF) [file pone.0280203.s006.tif]
